# Supplementary figures and images for: Mannan detecting C-type lectin receptor probes recognise immune epitopes with diverse chemical, spatial and phylogenetic heterogeneity in fungal cell walls
Source: PLoS Pathog. 2020 Jan 30;16(1):e1007927. doi: 10.1371/journal.ppat.1007927 (PMC7012452; doi:10.1371/journal.ppat.1007927)

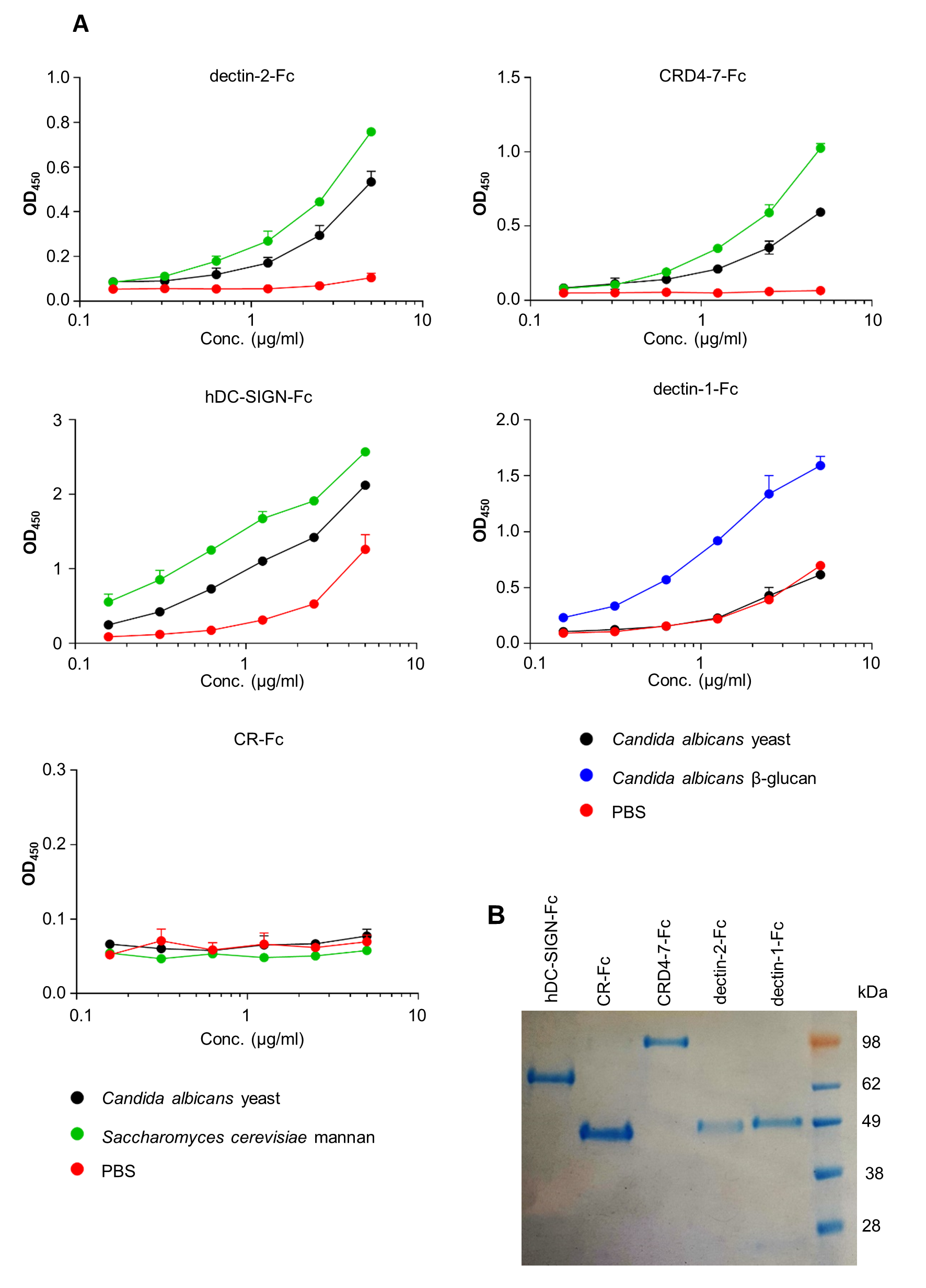

Supplement: S1 Fig — Purified Fc-lectin probes were screened against whole C. albicans (SC5314) yeast cells (black), purified cell wall mannan (green) or purified yeast β-glucan (blue) (A). Fc-lectin integrity was checked via reducing SDS-Page, expected band sizes were dectin-1-Fc 55 kDa, dectin-2-Fc 55 kDa, CRD4-7-Fc 110 kDa, CR-Fc 50 kDa, hDC-SIGN-Fc 69kDa (B). (TIF) [file ppat.1007927.s001.tif]

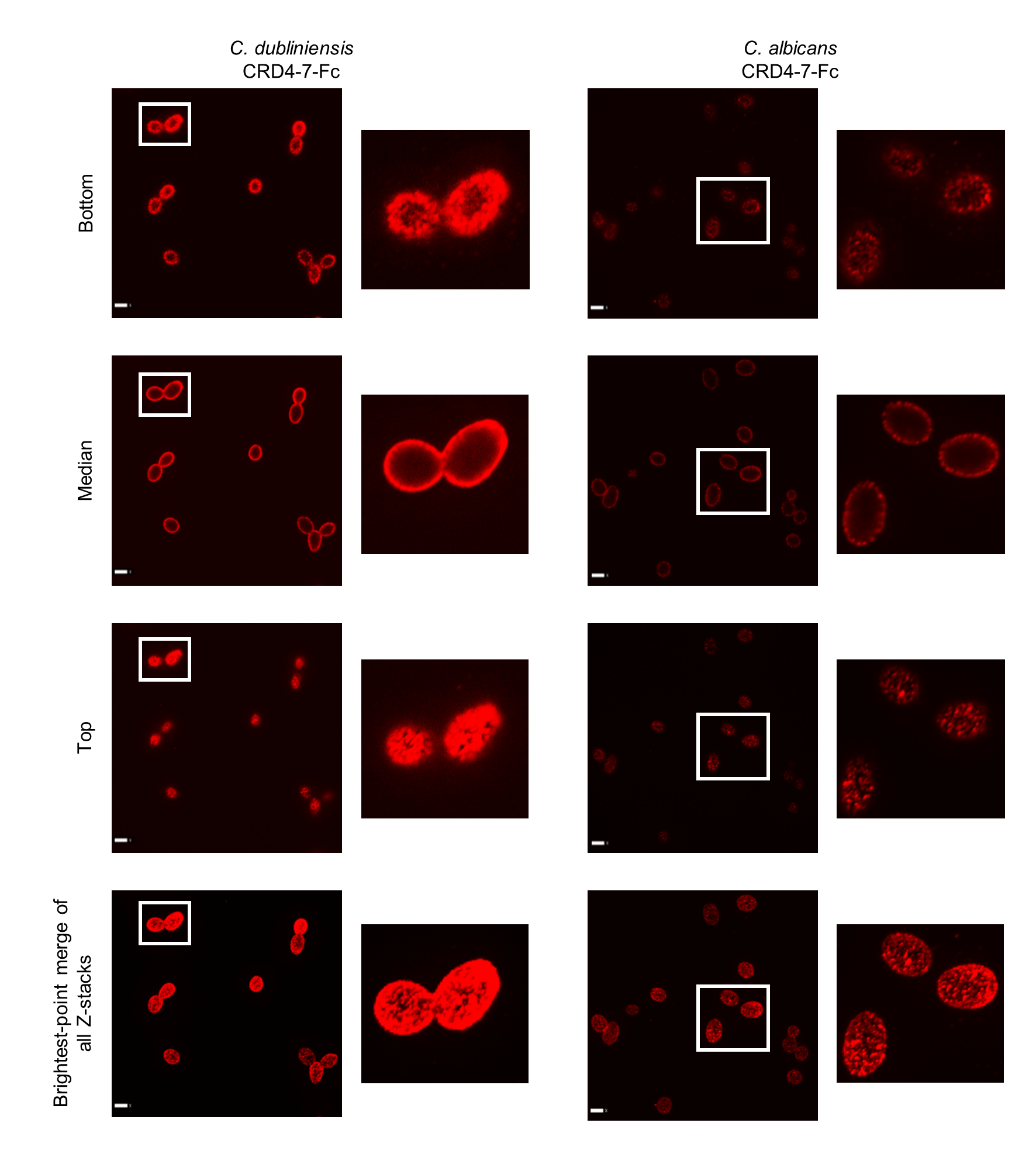

Supplement: S2 Fig — Images display Z-stack slices from the bottom, median and top as well as a merge of all Z-slices at the brightest point of CRD4-7-Fc binding to C. dubliniensis and C. albicans yeast cells. Images were generated using an UltraView VoX spinning disk confocal microscope and extended focus reconstructions in the Volocity software (Quorum Technologies) were utilised to generate a single image derived from the brightest-point merge of all the Z-slices through a cell. (TIF) [file ppat.1007927.s002.tif]

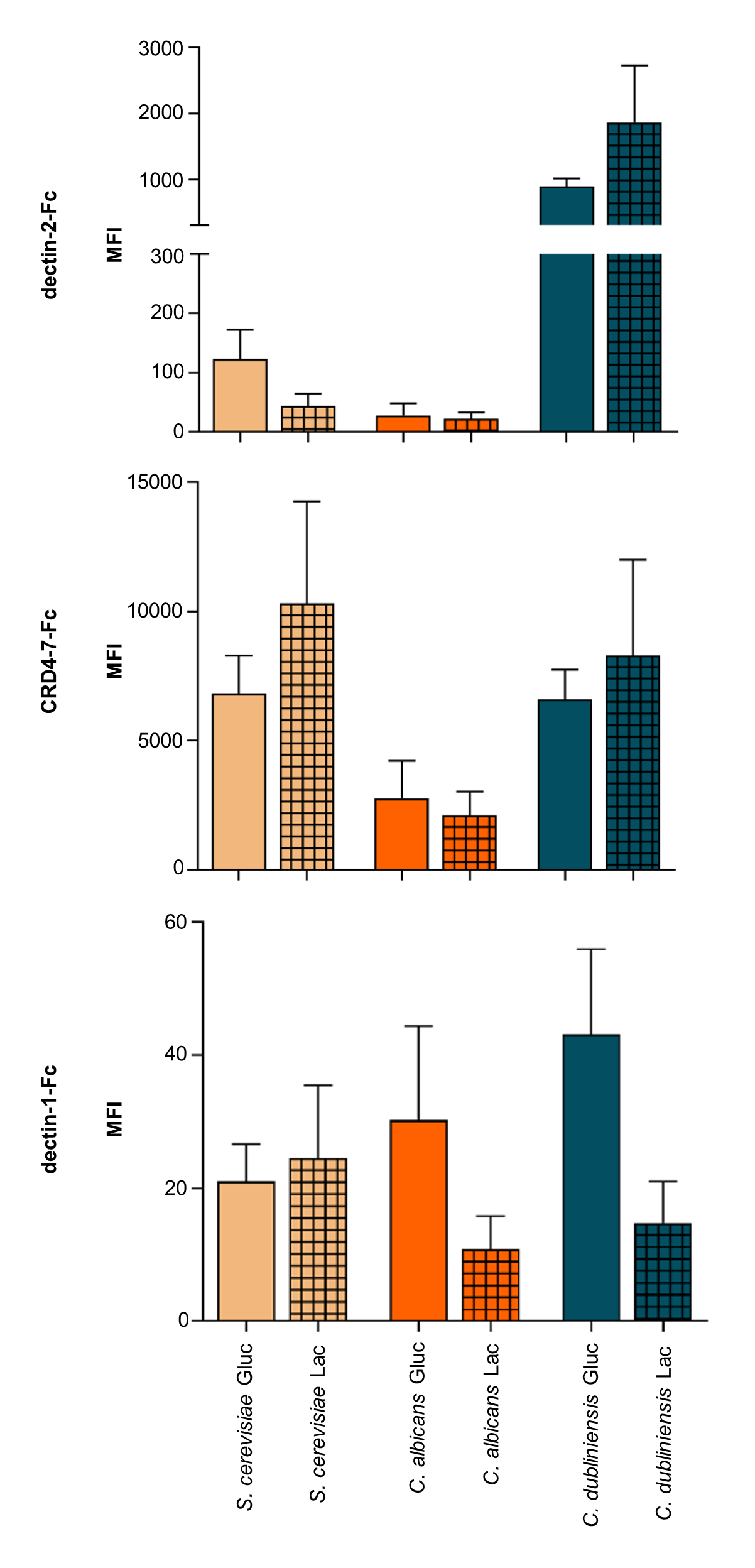

Supplement: S3 Fig — Indirect immunofluorescence staining of S. cerevisiae (NCPF8313), C. albicans (SC5314) and C. dubliniensis (WÜ284) yeast cells by dectin-2-Fc, CRD4-7-Fc and dectin-1-Fc represented as Median Fluorescent Intensity (MFI). 2.5 x 10^6^ cells were used in each analysis. The data represent three independent biological experiments ± SEM. Samples were analysed using a BD Fortessa flow cytometer, where 10,000 events were recorded for each sample (n = 30,000). Gluc, Glucose; Lac, Lactate. There were no statistically significant differences observed. (TIF) [file ppat.1007927.s003.tif]
