# Supplementary material for: Mannan detecting C-type lectin receptor probes recognise immune epitopes with diverse chemical, spatial and phylogenetic heterogeneity in fungal cell walls
Source: PLoS Pathog. 2020 Jan 30;16(1):e1007927. doi: 10.1371/journal.ppat.1007927 (PMC7012452; doi:10.1371/journal.ppat.1007927)
Supplement: S1 Table — (PDF) [file ppat.1007927.s004.pdf]

S1 Table. Fungal strains

Strain name	Genotype	Reference
C. albicans CAI4+CIp10 (NGY152)	*ura3Δ:: Δimm434/ura3Δ:: Δimm434 RPS1/rps1::URA3*	[1]
C. albicans BWP17 + CIp30	*ura3::imm434/ura3::imm434 his1::hisG/his1::hisG arg4::hisG/arg4::hisG*	[2, 3]
C. albicans SC5314	Clinical isolate	[4]
C. albicans Ysu751	Clinical isolate	[5]
C. albicans J990102	Clinical isolate	[5]
C. albicans IHEM3742	Clinical isolate	[5]
C. albicans AM2003/0069	Clinical isolate	[5]
C. albicans HUN92	Clinical isolate	[5]
C. glabrata SCS71182B	Clinical isolate	[6]
C. tropicalis AM2005/0546	Clinical isolate	Clinical isolate from Aberdeen Royal Infirmary
C. krusei SCS71987M	Clinical isolate	[6]
C. parapsilosis ATCC22019	Clinical isolate	[7]
C. dubliniensis Wü284	Clinical isolate	[8]
S. cerevisiae NCPF8313	Clinical isolate	Mycology Ref Lab, Bristol
mnn4Δ CDH15	*ura3Δ:: Δimm434/ura3Δ:: Δimm434 mnn4Δ::hisG/mnn4Δ::hisG, RPS1::URA3*	[9]
mnn2-26Δ NGY600	*mnn21::dpi200/mnn21::dpi200 mnn24::dpi200/mnn24::dpi200 mnn26::dpi200/mnn26::dpi200 mnn22::dpi200/mnn22::dpi200 mnn2::dpi200/mnn2::dpi200 mnn23::dpi200/mnn23::dpi200 ura3::imm434/ura3::imm434 rps1::CIp10/RPS1*	[10]
och1Δ NGY357	*och1Δ/och1Δ RPS1/rps1Δ::CIp10*	[11]
pmr1Δ NGY355	*ura3Δ::imm434/ura3Δ::imm434; pmr1Δ::hisG/pmr1Δ::hisG RPS1/rps1Δ::URA3*	[12]

Supplementary References for fungal strains

1. Brand A, MacCallum DM, Brown AJ, Gow NA, Odds FC. Ectopic expression of URA3 can influence the virulence phenotypes and proteome of *Candida albicans* but can be overcome by targeted reintegration of URA3 at the RPS10 locus. Eukaryot Cell. 2004;3(4):900-9. doi: 10.1128/EC.3.4.900-909.2004. PubMed PMID: 15302823; PubMed Central PMCID: PMCPMC500875.

2. Wilson RB, Davis D, Mitchell AP. Rapid hypothesis testing with *Candida albicans* through gene disruption with short homology regions. J Bacteriol. 1999;181(6):1868-74. PubMed PMID: 10074081; PubMed Central PMCID: PMCPMC93587.

3. Dennison PMJ, Ramsdale M, Manson CL, Brown AJP. Gene disruption in *Candida albicans* using a synthetic, codon-optimised Cre-loxP system. Fungal Genetics and Biology. 2005;42(9):737-48. doi: https://doi.org/10.1016/j.fgb.2005.05.006.

4. Gillum AM, Tsay EY, Kirsch DR. Isolation of the *Candida albicans* gene for orotidine-5'-phosphate decarboxylase by complementation of S. cerevisiae ura3 and *E. coli* pyrF mutations. Mol Gen Genet. 1984;198(2):179-82. PubMed PMID: 6394964.

5. MacCallum DM, Castillo L, Nather K, Munro CA, Brown AJ, Gow NA, et al. Property differences among the four major *Candida albicans* strain clades. Eukaryot Cell. 2009;8(3):373-87. Epub 2009/01/16. doi: 10.1128/EC.00387-08. PubMed PMID: 19151328; PubMed Central PMCID: PMCPMC2653250.

6. Odds FC, Hanson MF, Davidson AD, Jacobsen MD, Wright P, Whyte JA, et al. One year prospective survey of *Candida* bloodstream infections in Scotland. J Med Microbiol. 2007;56(Pt 8):1066-75. doi: 10.1099/jmm.0.47239-0. PubMed PMID: 17644714; PubMed Central PMCID: PMCPMC2884937.

7. Rudek W. Esterase activity in *Candida* species. J Clin Microbiol. 1978;8(6):756-9. PubMed PMID: 370150; PubMed Central PMCID: PMCPMC275337.

8. Jackson AP, Gamble JA, Yeomans T, Moran GP, Saunders D, Harris D, et al. Comparative genomics of the fungal pathogens *Candida dubliniensis* and *Candida albicans*. Genome Res. 2009;19(12):2231-44. doi: 10.1101/gr.097501.109. PubMed PMID: 19745113.

9. Hobson RP, Munro CA, Bates S, MacCallum DM, Cutler JE, Heinsbroek SE, et al. Loss of cell wall mannosylphosphate in *Candida albicans* does not influence macrophage recognition. J Biol Chem. 2004;279(38):39628-35. Epub 2004/07/22. doi: 10.1074/jbc.M405003200. PubMed PMID: 15271989.

10. Hall RA, Bates S, Lenardon MD, Maccallum DM, Wagener J, Lowman DW, et al. The Mnn2 mannosyltransferase family modulates mannoprotein fibril length, immune recognition and virulence of *Candida albicans*. PLoS Pathog. 2013;9(4):e1003276. Epub 2013/04/25. doi: 10.1371/journal.ppat.1003276. PubMed PMID: 23633946; PubMed Central PMCID: PMCPMC3636026.

11. Bates S, Hughes HB, Munro CA, Thomas WP, MacCallum DM, Bertram G, et al. Outer chain *N*-glycans are required for cell wall integrity and virulence of *Candida albicans*. J Biol Chem. 2006;281(1):90-8. Epub 2005/11/01. doi: 10.1074/jbc.M510360200. PubMed PMID: 16263704.

12. Bates S, MacCallum DM, Bertram G, Munro CA, Hughes HB, Buurman ET, et al. *Candida albicans* Pmr1p, a secretory pathway P-type Ca2+/Mn2+-ATPase, is required for glycosylation and virulence. J Biol Chem. 2005;280(24):23408-15. Epub 2005/04/20. doi: 10.1074/jbc.M502162200. PubMed PMID: 15843378.
