# Supplementary material for: Mannan detecting C-type lectin receptor probes recognise immune epitopes with diverse chemical, spatial and phylogenetic heterogeneity in fungal cell walls
Source: PLoS Pathog. 2020 Jan 30;16(1):e1007927. doi: 10.1371/journal.ppat.1007927 (PMC7012452; doi:10.1371/journal.ppat.1007927)
Supplement: S2 Table — (PDF) [file ppat.1007927.s005.pdf]

S2 Table. Saccharide probes included in the Fungal and Bacterial Polysaccharide Array.

Position	Probe[a]	Predominant oligosaccharide sequence where known[b]
1	Dextran *L. mesenteroides*	α1,6-Glc
2	Pullulan *P. pullulans*	Mixed α1,4/α1,6-Glc
3	Curdlan[c] *Agrobacterium* sp.	β1,3-Glc
4	NSG *S. cerevisiae*	Linear β1,3-Glc backbone with occasional monoglucosyl β1,6-Glc branches
5	PGG *S. cerevisiae*	
6	Lentinan *L. edodes*	
7	Grifolan *G. frondosa*	β1,3-Glc backbone with highly ramified oligomeric branches
8	Barley β-glucan	Mixed β1,3/β1,4-Glc
9	Oat β-glucan	Mixed β1,3/β1,4-Glc
10	Lichenan	Mixed β1,3/β1,4-Glc
11	Pustulan *U. papullosa*	β1,6-Glc
12	Mannan *S. cerevisiae*	α1,6-Man backbone with oligomeric α1,2-, α1,3-Man branches [1]
13	*N*-Mannoprotein *C.albicans*	α1,6-Man backbone with oligomeric α1,2-, α1,3-, and β-1,2-Man branches [1-3]
14	Mannoprotein *A. fumigatus*	Mannose-rich [4][4]
15	Lipomannan *M. tuberculosis*	Linear α1,6-Man backbone with monomannosyl α1,2-Man branches [5]
16	Lipoarabinomannan *M. tuberculosis*	Linear α1,6-Man backbone with monomannosyl α1,2-Man branches and α1,5-Ara polymer branched at certain positions with α1-3,1-5-Ara residues, which in turn are terminated by β1,2-Ara and capped by α1,2-Man units [5]
17	Lipoarabinomannan *M. smegmatis*	Linear α1,6-Man backbone with monomannosyl α1,2-Man branches and α1,5-Ara polymer branched at certain positions with α1-3,1-5-Ara residues, which in turn are terminated by β1,2-Ara and capped by phospho inositol [5]
18	Native *O*-glycoprotein *M. tuberculosis*	α1,2-Man [6]
19	Glucurono-XyloMannan[d] *T. fuciformis*	α1,3-Man with Xyl, GlcA and Fuc branches
20	GN6-AO[e]	GlcNAcβ-4GlcNAcβ-4GlcNAcβ-4GlcNAcβ-4GlcNAcβ-4GlcNAc-AO

[a] Unless otherwise indicated the saccharide probes are polysaccharides: NSG, Neutral soluble β-glucan; PGG, Poly-(1,6)-D-glucopyranosyl-(1,3)-D-glucopyranose

[b] Glc, Glucose; Man, Mannose; Gal, Galactose; Ara, Arabinose; Xyl, xylose; GlcA, Glucuronic acid; Fuc, fucose; GlcNAc, *N*-acetylglucosamine.

[c,d] Curdlan polysaccharide was solubilized in 50mM NaOH and Glucurono-XyloMannan in 150 mM NaCl, prior printing.
[e] GN_6-AO, neoglycolipid (NGL) probe prepared from reducing hexasaccharide of chitin (β1,4-linked *N*-acetylglucosamine, GlcNAc) by oxime ligation with an aminooxy (AO) functionalised DHPE[7].

Supplementary References

1. Takahara K, Arita T, Tokieda S, Shibata N, Okawa Y, Tateno H, et al. Difference in fine specificity to polysaccharides of *Candida albicans* mannoprotein between mouse SIGNR1 and human DC-SIGN. Infection and immunity. 2012;80(5):1699-706. doi: 10.1128/IAI.06308-11. PubMed PMID: 22331432.

2. Masuoka J. Surface glycans of *Candida albicans* and other pathogenic fungi: physiological roles, clinical uses, and experimental challenges. Clin Microbiol Rev. 2004;17(2):281-310. doi: 10.1128/cmr.17.2.281-310.2004. PubMed PMID: 15084502.

3. Lowman DW, Ensley HE, Greene RR, Knagge KJ, Williams DL, Kruppa MD. Mannan structural complexity is decreased when *Candida albicans* is cultivated in blood or serum at physiological temperature. Carbohydrate research. 2011;346(17):2752-9. Epub 10/02. doi: 10.1016/j.carres.2011.09.029. PubMed PMID: 22030461.

4. Thornton CR. Development of an immunochromatographic lateral-flow device for rapid serodiagnosis of invasive aspergillosis. Clin Vaccine Immunol. 2008;15(7):1095-105. Epub 05/07. doi: 10.1128/CVI.00068-08. PubMed PMID: 18463222.

5. Mishra AK, Driessen NN, Appelmelk BJ, Besra GS. Lipoarabinomannan and related glycoconjugates: structure, biogenesis and role in *Mycobacterium tuberculosis* physiology and host-pathogen interaction. FEMS Microbiol Rev. 2011;35(6):1126-57. Epub 05/31. doi: 10.1111/j.1574-6976.2011.00276.x. PubMed PMID: 21521247.

6. Dobos KM, Khoo KH, Swiderek KM, Brennan PJ, Belisle JT. Definition of the full extent of glycosylation of the 45-kilodalton glycoprotein of *Mycobacterium tuberculosis*. J Bacteriol. 1996;178(9):2498-506. doi: 10.1128/jb.178.9.2498-2506.1996. PubMed PMID: 8626314.
