# Supplementary material for: Mannan detecting C-type lectin receptor probes recognise immune epitopes with diverse chemical, spatial and phylogenetic heterogeneity in fungal cell walls
Source: PLoS Pathog. 2020 Jan 30;16(1):e1007927. doi: 10.1371/journal.ppat.1007927 (PMC7012452; doi:10.1371/journal.ppat.1007927)
Supplement: S3 Table — A. List of glycan probes, fluorescence binding scores and relative binding intensities ('matrix') elicited with the Fc-lectins. B. List of glycan probes and their sequences in the screening array. (PDF) [file ppat.1007927.s006.pdf]

S3 Table A. List of glycan probes, fluorescence binding scores and relative binding intensities ('matrix') elicited with the Fc-lectins.

		Dectin-2-Fc		CRD4-7-Fc		DC-SIGN-Fc	
Pos[a]	Probe[b]	Score[c]	Error[d]	Score[c]	Error[d]	Score[c]	Error[d]
Lactose and N-acetyllactosamine-based							
1	Galactocerebrosides	-	399	-	246	-	59
2	H-Di	-	46	-	92	4,752	169
3	A-Tri	-	7	-	170	7,853	176
4	B-Tri	-	14	-	89	8,267	164
5	B-Tri-AO	-	50	5,465	44	16,967	399
6	GSC-426	-	171	-	8	62	44
7	Sulfatide	-	177	-	77	-	26
8	GSF-1	-	69	-	101	-	29
9	GSC-209	-	161	-	179	-	20
10	GSC-210	-	44	-	469	-	44
11	GSC-187	-	52	-	86	-	31
12	GSC-40	-	22	-	46	-	47
13	GSC-230	-	103	-	68	-	60
14	GSC-27	-	55	-	381	-	11
15	GSC-144	-	208	-	50	-	4
16	GSC-13	-	79	-	80	-	11
17	GSC-72	-	39	-	54	-	29
18	GSC-231	-	35	-	18	-	30
19	GSC-439	-	65	-	87	-	9
20	Glucocerebrosides	-	91	-	79	-	12
21	GSF-19	-	0	-	125	-	11
22	GSC-60	-	186	-	53	-	21
23	GSC-9	-	19	-	20	-	2
24	GSC-62	-	124	-	56	-	15
25	GSC-59	-	80	-	163	-	38
26	GSC-95	-	59	-	20	-	32
27	GSC-232	-	162	-	159	-	33
28	Lactocerebrosides	-	119	-	157	-	24
29	Lac	-	141	-	53	-	75
30	Lac-AO	-	120	-	36	-	4
31	GSC-432	-	73	-	11	-	16
32	GSC-296	-	144	-	54	-	2
33	GSC-353	717	135	1,373	255	3,238	283
34	GalNAcα-3Galβ-4Glc	-	191	-	82	-	27
35	Globotri-AO	-	385	-	13	267	53
36	Ceramide trihexoside	-	1	-	297	-	56
37	Globoside (P-antigen)	-	58	-	3	-	7
38	Forssmann glycolipid	-	65	-	116	-	5
39	Fuc(3)-Lac-AO	-	261	1,297	77	18,142	65
40	GSC-430	-	35	-	143	9,068	47
41	GSC-260	-	10	-	58	288	37

42	GSC-150	-	97		-	16		3,429	107
43	GSC-160	-	8		-	146		1,856	214
44	NeuAcα-(3')Lac	-	106		-	439		-	48
45	NeuAcα-(3')Lac-AO	-	140		-	11		6	20
46	Neu4,5Ac-(3')Lac	-	194		-	55		-	38
47	Neu4,5Ac-(3')Lac-AO	-	59		-	8		-	85
48	GSC-16	-	107		-	49		-	35
49	GSC-178	-	46		-	99		-	22
50	GSC-17	-	68		-	118		-	59
51	GSC-18	-	30		-	4		-	11
52	GSC-197	-	200		-	40		-	34
53	GSC-199	-	202		-	57		-	3
54	GSC-198	-	75		-	100		-	90
55	GSC-75	-	60		-	15		-	8
56	GSC-76	-	107		-	383		-	64
57	GSC-77	-	24		-	34		-	20
58	GSC-153	-	235		-	49		-	44
59	GSC-51	-	154		-	138		-	63
60	GSC-78	-	184		-	321		-	46
61	GSC-79	-	273		-	119		-	24
62	GSC-23	-	5		-	39		-	35
63	GSC-24	-	27		-	63		-	20
64	GSC-50	-	84		-	42		-	17
65	GSC-229	-	313		-	1		-	44
66	GSC-96	-	15		-	97		-	29
67	GSC-437	-	241		-	28		-	6
68	Neuα-(3')Lac	-	50		-	319		-	33
69	Neuα-(3')Lac-AO	-	100		-	19		-	8
70	NeuAcα-(6')Lac	-	40		-	278		-	50
71	NeuAcα-(6')Lac-AO	-	98		-	56		-	54
72	GSC-61	-	178		-	154		-	2
73	GSC-12	-	70		-	290		-	60
74	GSC-234	-	43		-	116		166	13
75	GSC-73	-	85		-	613		-	25
76	Neuα-(6')Lac	-	42		-	308		-	20
77	Neuα-(6')Lac-AO	-	75		-	106		-	10
78	NeuAcβ-(3')Lac	-	210		-	119		-	101
79	NeuAcβ-(3')Lac-AO	-	106		-	7		-	29
80	NeuAcβ-(6')Lac	-	130		-	196		-	8
81	NeuAcβ-(6')Lac-AO	-	125		-	25		-	51
82	GSC-161	-	30		-	60		71	66
83	GSC-162	-	131		-	2		42	33
84	LacNAc(1-3)	-	104		-	13		-	8
85	LacNAc(1-3)-AO	-	53		-	41		-	5
86	LacNAc	-	124		-	41		-	31
87	LacNAc-AO	-	231		-	17		-	19

88	Galα-4Galβ-4GlcNAc	-	41	-	74	-	1
89	SU(3')-LN	-	129	-	104	184	52
90	Lea-Tri	-	30	42	19	15,485	196
91	Lea-Tri-AO	-	176	43	191	15,709	447
92	Lex-Tri	-	81	-	121	165	32
93	Lex-Tri-AO	-	69	128	220	12,667	272
94	Lex-Tri-(Me)AO	-	67	-	248	12,182	195
95	SU(3')-Lea-Tri	-	8	843	70	10,637	351
96	SU(3')-Lex-Tri	-	68	-	118	1,232	69
97	NeuAcα-(3')LN	-	32	-	51	-	44
98	NeuAcα-(3')LN-AO	-	19	-	51	-	8
99	PI-1	-	40	-	21	-	50
100	PI-1-AO	-	152	-	116	0	0
101	PI-2	-	5	-	39	-	40
102	PI-2-AO	-	110	-	177	686	2
103	NeuAcα-(6')LN	-	53	-	268	-	14
104	NeuAcα-(6')LN-AO	-	28	-	72	-	32
105	Neu5,9Ac-(6')LN	-	11	-	336	-	62
106	SA(3')-Lea-Tri	-	29	-	1	2,546	92
107	SA(3')-Lea-Tri-AO	-	103	-	44	6,090	546
108	SA(3')-Lex-Tri	-	28	-	305	-	1
109	SA(3')-Lex-Tri-AO	-	377	-	257	2,240	229
110	GSC-440	-	59	-	27	-	30
111	GSC-512	-	214	-	149	-	5
112	GSC-513	-	95	-	10	82	12
113	GSC-511	-	17	-	172	-	14
Lacto-N-neotetraose and Lacto-N-tetraose-based							
114	GSC-225	-	157	-	38	2,257	568
115	GSC-236	-	54	-	110	4,796	538
116	GSC-479	-	389	-	89	305	102
117	GSC-105	-	195	-	273	-	10
118	GSC-121	-	196	-	90	-	27
119	GSC-123	-	529	-	148	-	43
120	GSC-133	-	48	-	134	-	32
121	GSC-131	-	94	-	97	-	47
122	GSC-163	-	1	-	31	-	73
123	GSC-127	-	274	-	81	-	34
124	GSC-341	-	82	-	61	2,599	180
125	GSC-177	-	11	-	78	426	7
126	GSC-175	-	44	-	135	-	42
127	GSC-176	-	347	-	73	-	96
128	GSC-257	-	110	-	61	-	11
129	DLNN	-	81	-	2	5,209	78
130	LNT	-	6	-	28	283	51
131	Paragloboside	-	44	-	87	-	34
132	LNnT	-	20	-	267	-	15

133	B-like pentaosylceramide	-	131	-	72	-	44
134	Klaus glycolipid	-	19	-	46	-	2
135	GSC-207	-	87	-	84	-	32
136	GSC-191	-	55	-	57	-	9
137	GSC-189	-	259	-	327	-	97
138	SU(3')-Tri	-	5	-	144	-	55
139	GSC-208	-	65	-	62	-	66
140	GSC-192	-	72	-	130	-	61
141	GSC-190	-	19	-	88	-	288
142	Led-II pentaosylceramide	-	64	-	114	16	6
143	Led-I pentaosylceramide	-	271	-	45	-	9
144	LNFP-I	-	21	-	203	1,408	59
145	B-hexaosylceramide	-	256	-	173	204	1
146	A-Hexa	-	14	-	73	-	47
147	A-Hepta	-	32	-	218	15,235	1,124
148	LNFP-II	-	21	2,463	167	21,264	1,448
149	LNDFH-II	-	222	1,865	343	22,044	1,360
150	Leb-hexaosylceramide	-	51	-	180	11,755	1,340
151	LNDFH-I	-	161	377	87	27,665	954
152	LNTFH-I	-	71	-	339	26,102	374
153	LNFP-III	-	123	-	62	12,583	27
154	LNFP-III-AO	-	69	-	305	22,462	1,633
155	LNnDFH-I	-	81	-	271	21,581	2,042
156	LNnDFH-II	-	98	-	305	21,195	27
157	LNnDFH-V	-	134	-	138	14,281	392
158	LNnTFH-I	-	187	-	88	15,768	517
159	SU(3')-LNFP-II	-	26	1,900	177	23,452	1,933
160	SU(6')-LNFP-II	-	3	1,856	251	19,534	2,362
161	SU(3')-LNFP-III	-	88	-	228	13,535	193
162	SU(6')-LNFP-III	-	30	-	110	1,140	140
163	SU(3',6)-LNFP-III	-	35	-	120	454	113
164	LSTa	-	36	-	72	-	84
165	GSC-272	-	97	-	204	-	17
166	GSC-147	-	6	-	50	-	6
167	GSC-396	-	216	-	181	-	2
168	LSTb	-	216	-	252	240	65
169	GSC-397	-	224	-	111	-	10
170	DSLNT	-	68	-	5	-	35
171	Sialylparagloboside	-	8	-	492	-	16
172	GSC-273	-	53	-	92	-	22
173	GSC-31	-	390	-	22	-	14
174	LSTc	-	277	-	27	-	58
175	GSC-516B	-	45	-	17	-	2
176	SA(3/6)LNFP-I	-	114	-	93	11,405	147
177	SA(3')-LNFP-II	-	185	-	42	10,406	665
178	SA(6')-LNFP-VI	-	54	-	117	115	65

179	GSC-533	-	86	-	305	1,000	687
180	GSC-64	-	171	-	215	1,597	884
181	SA(3')-LNFP-III	2,598	162	201	2	6,216	631
182	GSC-472	-	170	-	74	396	56
183	GSC-97	-	68	-	113	455	20
184	GSC-314	-	53	-	60	5,038	176
185	GSC-149	-	141	-	25	4,417	54
186	GSC-311	-	35	-	49	-	94
187	GSC-268	-	30	-	7	-	39
188	GSC-268 deNAc	-	153	-	67	-	27
189	GSC-269	-	3	-	109	1,818	36
190	GSC-406	-	67	-	69	326	38
191	GSC-270	-	186	-	54	-	0
Polylactosamine Ii-active							
192	pLNnH	-	90	-	44	-	109
193	GSC-216	-	114	-	171	-	17
194	GSC-217	-	184	-	79	-	47
195	GSC-218	-	4	-	57	-	66
196	GSC-219	-	193	-	1	-	84
197	LNH	-	610	-	97	464	32
198	iLNO	-	175	-	166	21	44
199	LND	-	12	-	91	-	54
200	LNnH	-	14	-	19	-	20
201	Nonaosylceramide	-	41	-	280	2,122	450
202	I-octaosylceramide	-	82	-	36	-	58
203	I-dodecaosylceramide	-	103	-	101	1,117	52
204	I-hexadecaosylceramide	-	173	-	53	-	79
205	I-eicosaosylceramide	-	33	-	121	-	42
206	B-like decaosylceramide	-	87	-	98	-	51
207	B-like pentadecaosylceramide	-	134	-	193	-	57
208	B-like eicosaosylceramide	-	24	-	67	-	39
209	B-like pentaeicosaosylceramide	-	169	-	233	-	17
210	pLNFH-IV	-	52	-	19	4,737	327
211	DFpLNH-II	-	55	-	13	7,723	451
212	TFpLNH-I	-	86	-	201	14,625	195
213	MFLNH-III	-	68	-	173	12,602	369
214	DFLNH(b)	-	70	1,896	53	21,792	519
215	DFLNH(c)	-	73	-	161	11,318	459
216	DFLNH(a)	-	1	-	93	17,789	613
217	TFLNH	-	95	612	44	19,010	1,508
218	MFiLNO-IV	-	137	-	37	6,118	35
219	TFiLNO	-	99	468	84	9,719	1,230
220	MFLND	-	22	-	30	1,957	319
221	MFLNnH(a)	-	24	-	118	14,400	907
222	DFLNnH	-	27	-	93	12,724	514
223	B-III dodecaosylceramide	-	87	-	282	1,251	227

224	B-IV tetradecaosylceramide	-	124	-	79	242	96
225	MSLNH	-	71	-	103	211	2
226	MSLNnH-I	-	22	-	41	-	102
227	DSLNnH	-	6	-	16	-	2
228	MSMFLNH	-	15	-	6	10,624	194
229	MFMSLNnH	-	258	-	52	9,389	571
230	GSC-221	-	331	-	201	569	171
231	GSC-220	-	122	-	67	2,110	191
232	C4U	-	68	-	131	-	58
N-glycans							
233	Man2(α2)	-	6	44	74	9,901	697
234	Man2(α3)	-	113	-	52	-	68
235	Man2(α6)	-	5	389	236	11,000	317
236	Man3(α3,α6)	-	38	2,155	260	15,043	381
237	Man5(α3,α6)	-	145	7,948	459	20,959	909
238	Man1GN1	-	141	2,943	228	11,097	658
239	Man2GN1	-	112	832	169	7,319	480
240	Man2aGN2	-	133	5,612	69	8,093	385
241	Man3GN2	-	4	6,619	1,221	11,531	214
242	Man4aGN2	-	0	7,372	414	17,502	898
243	Man4bGN2	-	480	12,285	747	15,760	111
244	Man5GN2	-	62	6,444	456	19,125	917
245	Man6GN2	-	5	7,260	891	19,587	149
246	Man7(D1)GN2	498	408	7,710	1,160	21,084	2,259
247	Man7(D1)GN2-AO	960	98	7,266	715	20,945	360
248	Man7(D3)GN2	-	182	6,212	348	16,911	2,494
249	Man8(D1D3)GN2	731	100	7,553	1,729	21,157	1,137
250	Man9GN2	1,731	152	5,893	605	24,094	1,165
251	Man9GN2-AO	1,218	313	6,722	334	20,171	812
252	Glc1Man9GN2	-	341	2,779	97	20,696	1,268
253	Glc1Man9GN2-AO	-	109	2,316	412	18,860	105
254	Glc2Man7(D1)GN1-AO	-	61	269	478	9,700	686
255	Glc3Man7(D1)GN1-AO	-	89	300	59	8,683	18
256	Man3XylGN2	-	187	336	16	11,095	1,132
257	N1	-	69	484	118	7,116	28
258	N2	-	52	-	70	3,849	329
259	N4	-	29	553	47	5,030	660
260	GlcNac2Man3-AO	-	7	-	31	11,919	995
261	N3	-	148	-	104	8,844	904
262	NGA2	-	82	104	80	7,558	19
263	NGA2B	-	56	-	65	4,481	492
264	NGA3B	-	211	-	32	9,738	355
265	NGA4	-	19	682	27	10,676	541
266	NGA5B	-	5	163	286	8,415	259
267	GNMan5BGN2	-	15	2,469	523	16,145	116
268	NA2	-	149	-	41	862	26

269	NA3	-	118	-	71	-	7
270	NA4	-	62	-	37	-	46
271	Fuc-GlcNAc	-	142	7,290	945	21,331	535
272	Man3FGN2	-	37	8,788	39	14,944	306
273	Man3FXylGN2	-	55	1,700	199	16,064	707
274	NGA2F	-	156	31	40	7,881	298
275	NA2F	-	71	-	59	276	94
276	NA2F-AO	-	269	-	92	1,527	160
277	NA2FB	-	85	-	52	1,002	43
278	NA3-Lex	-	99	-	32	6,229	846
279	A2(2-6)	-	49	-	40	-	10
280	AGP-Bi-Ac2	-	57	-	51	-	28
281	AGP-Bi-Gc2	-	32	-	34	-	62
282	AGP-Bi-AcGc	-	177	-	19	-	13
283	A3	-	264	-	108	-	23
284	A2F(2-3)	-	246	-	159	-	19
Gangliosides							
285	GM4	-	84	-	122	-	32
286	SM3	-	12	-	89	102	38
287	Haematoside	-	212	-	15	-	3
288	GM3	-	58	-	54	-	42
289	GM3(Gc)	-	88	-	352	-	37
290	Asialo-GM2	-	62	-	19	-	28
291	SB2	-	66	-	357	2,082	60
292	GM2	-	23	-	44	-	37
293	GSC-576	-	26	-	75	-	15
294	GSC-108	-	165	-	56	-	70
295	GSC-193	-	245	-	51	-	27
296	Asialo-GM1	-	58	-	70	-	21
297	Asialo-GM1-Tetra	-	57	-	58	-	14
298	SM1a	-	127	-	18	-	15
299	SB1a	-	29	-	2	597	45
300	GSC-335	-	212	-	206	-	7
301	GM1	-	98	-	189	-	6
302	GM1-penta	-	121	-	2	-	24
303	GM1(Gc)	-	88	-	54	-	9
304	GM1(Gc)-penta	-	45	-	12	-	12
305	GD1a	-	40	-	66	-	20
306	GD1a-hexa	-	100	-	123	-	43
307	GalNAc-GD1a(Ac,Gc)	-	23	-	82	-	32
308	GSC-195	-	53	-	154	-	52
309	GD3	-	58	-	5	-	21
310	GD3-tetra	-	51	-	48	-	7
311	GD3-tetra-AO	-	364	-	50	-	36
312	GD2	-	146	132	47	-	67
313	GD1b	-	29	-	36	-	12

314	GD1b-DH	-	129	-	31	-	2
315	GT1a	-	358	-	230	-	3
316	GT1b	-	273	-	21	-	13
317	GQ1b	-	88	-	25	-	13
318	GSC-442	-	38	-	84	-	17
319	GSC-68	-	57	-	8	-	27
320	GSC-107	-	18	-	206	-	15
321	GSC-118	-	297	-	86	-	4
O-glycans							
322	GalNAc-Ser	-	5	-	175	-	2
323	GalNAc-Thr	-	117	-	50	-	7
324	BSM-Di-A1-AO	-	20	-	89	-	6
325	BSM-Di-A2-AO	-	175	-	49	-	0
326	GalNAcα-3GalNAc	-	193	-	85	23	37
327	Galβ-3GalNAc	-	287	-	59	181	24
328	Galβ-3GalNAc-AO	-	49	-	17	799	65
329	Galβ-6GalNAc	-	18	-	128	-	1
330	Galβ-6GalNAc-AO	-	166	-	32	-	4
331	Man-Ser	-	152	-	30	9,919	57
332	Man-Ser-Succ	-	143	-	64	3,055	352
333	Man-Thr	-	183	341	143	11,365	98
334	Man-Thr-Succ	-	222	-	145	727	94
335	A8/1	-	141	-	100	153	15
336	A8/2	-	41	-	68	9,810	340
337	A15/1	-	176	-	205	-	34
338	A15/3	-	85	1	74	13,265	865
339	Notch-1	-	47	310	119	15,615	347
340	Notch-2	-	33	-	32	658	43
341	Notch-3	-	89	-	82	-	48
342	GSC-488	-	42	-	102	-	6
343	GSC-491	-	113	-	100	-	18
344	GSC-489	-	37	-	154	-	16
345	DST	-	94	-	47	-	51
346	DST-AO	-	51	-	112	-	47
347	GSC-490	-	158	-	112	-	9
348	GlcNAcβ-3Fuc-AO	-	30	-	57	2,809	348
349	GlcNAcβ1-2Fuc-AO	-	336	-	24	3,493	52
350	GlcNAcβ1-4Fuc-AO	-	13	-	53	7,917	17
351	GlcNAcβ-2Man-AO	-	53	-	373	7,165	94
Polysialyl							
352	SA2(α8)	-	156	-	12	-	17
353	SA3(α8)	-	51	-	5	-	35
354	SA4(α8)	-	138	-	23	-	43
355	SA5(α8)*	-	149	-	110	-	5
356	SA6(α8)*	-	202	-	34	-	18
357	SA7(α8)*	-	107	-	32	-	65

358	SA8(α8)*	-	34		-	41	-	7
359	SA9(α8)*	-	4		-	43	-	25
360	SA10(α8)*	-	13		-	188	-	31
Glycosaminoglycans								
361	HA-S4*	-	35		-	84	-	86
362	HA-S14*	-	125		-	167	-	21
363	Hep-Di IS	2,082	35		2,223	218	5,163	693
364	Hep-Di-IS-AO	592	134		667	260	3,803	445
365	CSA-4*	-	44		-	70	645	110
366	CSA-14*	-	7		-	56	-	34
367	CSB-4*	-	586		-	262	852	88
368	CSB-14*	-	78		597	374	1,322	94
369	CSC-4*	-	57		-	117	273	20
370	CSC-14*	-	16		1,063	69	916	74
371	Hep-4-AO*	1	182		365	75	3,082	160
372	Hep-14-AO*	-	99		-	18	721	86
373	HS-S4-AO*	-	2		291	267	2,682	108
Homo-oligomers								
374	GN2-AO	-	293		-	53	3,250	2
375	GN3	200	82		59	34	4,423	187
376	GN3-AO	-	152		87	6	7,639	111
377	GN4-AO*	-	60		1,594	961	6,622	94
378	GN5-AO*	-	113		-	179	6,744	221
379	GN7-AO*	-	108		-	4	2,087	42
380	GN8-AO*	-	26		-	9	1,063	25
381	Man4(β4)	-	67		8,201	156	16,780	411
382	Man6(β4)	-	33		9,740	1,224	14,169	613
383	Xyl5(β4)	-	44		-	18	1,401	18
384	Xyl6(β4)	-	7		-	89	181	56
385	Ara6(α5)	-	43		-	25	2	29
386	Ara7(α5)	-	98		-	283	-	20
387	Nigerose-AO	-	83		-	27	8,586	198
388	Malto-2-AO	-	300		-	177	6,061	95
389	Malto-4-AO	-	169		-	42	6,399	4
390	Malto-6-AO	-	60		-	168	7,936	19
391	Malto-7-AO	-	242		-	62	8,173	380
392	Malto-8-AO*	-	1		-	136	8,589	36
393	Malto-9-AO*	-	121		-	47	4,510	211
394	Malto-10-AO*	-	442		-	204	4,086	2,607
395	Malto-11-AO*	-	58		-	28	4,638	332
396	Malto-12-AO*	-	202		-	71	7,487	660
397	Malto-13-AO*	-	633		8	389	2,298	960
398	Dext-2-AO	-	24		-	184	4,809	501
399	Dext-3-AO	-	241		-	365	5,054	197
400	Dext-4-AO	-	75		-	94	4,525	319
401	Dext-5-AO*	-	252		-	86	4,797	38

402	Dext-6-AO*	-	317	-	77	5,933	14
403	Dext-8-AO*	-	32	97	11	3,749	25
404	Dext-9-AO*	-	107	-	86	4,775	91
405	Dext-10-AO*	-	14	-	73	2,108	163
406	Dext-11-AO*	-	366	-	54	1,902	153
407	Dext-12-AO*	-	104	-	100	2,101	38
408	Dext-13-AO*	-	297	-	23	2,171	109
409	Lam-2-AO	-	85	-	93	10,239	38
410	Lam-3-AO	-	296	-	3	7,835	571
411	Lam-4-AO	-	369	110	11	9,665	269
412	Lam-5-AO	-	205	414	71	8,452	231
413	Lam-6-AO*	-	88	370	502	8,981	73
414	Lam-7-AO	-	335	188	192	2,738	441
415	Curd-8-AO*	276	97	335	48	5,905	373
416	Curd-9-AO*	-	143	-	74	4,489	173
417	Curd-10-AO*	-	76	-	118	4,629	95
418	Curd-11-AO*	-	146	183	65	2,624	728
419	Curd-12-AO*	10	24	141	44	4,918	770
420	Curd-13-AO*	-	424	-	133	2,082	210
421	Cello-3-AO	-	65	-	57	9,217	221
422	Cello-4-AO	-	78	85	109	5,184	415
423	Cello-5-AO*	-	279	-	76	4,492	1,234
424	Cello-6-AO*	-	46	-	527	4,180	63
425	Cello-7-AO*	-	73	196	40	4,298	60
426	Cello-8-AO*	-	218	116	27	4,241	237
427	Cello-9-AO*	-	108	-	47	7,012	239
428	Cello-10-AO*	-	9	146	297	6,514	239
429	Cello-11-AO*	-	237	185	4	6,518	90
430	Cello-12-AO*	-	308	98	67	5,214	140
431	Cello-13-AO*	-	52	159	41	5,025	628
432	Pust-3-AO	-	149	3	46	9,811	339
433	Pust-4-AO	-	348	-	17	9,108	395
434	Pust-5-AO	-	140	-	260	9,862	521
435	Pust-6-AO	-	360	33	50	5,572	1,608
436	Pust-7-AO*	-	359	-	51	6,897	441
437	Pust-8-AO*	-	300	-	304	4,122	805
Miscellaneous							
438	Gal	-	177	-	61	-	38
439	Gal-AO	-	116	-	95	-	36
440	GalNAc	-	162	-	85	119	50
441	GalNAc-AO	-	96	-	59	-	47
442	Glc	-	80	-	111	-	74
443	Glc-AO	-	120	-	102	4,593	52
444	GN	-	168	-	32	-	39
445	GN-AO	-	133	-	91	382	27
446	Man-AO	-	15	-	9	6,242	917

447	Fuc	-	166	-	36	87	85
448	Fuc-AO	-	242	-	197	337	32
449	NeuAc	-	78	-	11	114	61
450	NeuAc-AO	-	174	-	45	94	23
451	NeuGc	-	37	-	85	-	9
452	NeuGc-AO	-	209	-	221	-	52
453	Rha	-	56	-	116	-	41
454	Rha-AO	-	59	-	97	-	42
455	Galα-6Glc-AO	-	223	-	114	670	348
456	(6P)-Glc-AO	-	7	-	89	-	17
457	(6P)-Man	-	123	-	145	6	38
458	(6P)-Man-AO	-	27	-	45	-	17
459	(6P)-Fructose-AO	-	110	-	100	-	16
460	SU-Tyr	3,714	160	3,151	30	7,042	319
461	SU-Cholesterol	-	9	-	35	-	24
462	GN-Asn	-	180	-	56	8,669	338
463	Xyl3Glc4	-	114	-	53	1,338	12
464	GSC-284	-	108	-	94	-	61
465	GSC-575	-	375	-	137	-	24
466	GSC-70	-	55	-	80	-	32
467	GSC-154	-	42	-	160	-	43
468	GSC-446	-	18	-	33	-	6
469	GSC-441	-	88	-	70	-	7
470	GSC-384	-	127	-	130	969	22
471	Glc4(α6,α4,α4)	-	8	-	45	2,809	20
472	Glc(α6,α4,α4)-AO	-	110	-	21	5,170	58
473	O1-AO	-	12	-	221	12,605	455
474	Rutinose-AO	-	27	-	64	179	89

Asterisks that follow the names of certain probes indicate that predominant components are shown.

[a] Pos, Probe position in the binding chart.
[b] The glycan probes are all lipid-linked glycans, neoglycolipids (NGLs), natural and synthetic glycolipids. These are from the collection assembled in the course of research in the Glycosciences Laboratory. DH, NGLs prepared from reducing oligosaccharides by reductive amination with the amino lipid, 1,2-dihexadecyl-*sn*-glycero-3-phosphoethanolamine (DHPE), DH-NGLs; AO, NGLs prepared from reducing oligosaccharides by oxime ligation with an aminooxy (AO) functionalised DHPE; Cer, natural glycolipids with various ceramide moieties; Cer36 and Cer42, synthetic glycolipids with ceramide having a total of 32 and 42 carbon atoms, respectively; C30, a synthetic lipid [2-(tetradecyl)hexadecanol] with 30 carbon atoms. This is a sub-set of a larger array that will be described elsewhere.
[c] Fluorescence binding signals are shown as means of duplicate spots at 5 fmol per spot; -, indicates fluorescence intensity less than 1.
[d] Half of the difference of signal intensities of duplicated spots of each glycan probe.

S3 Table B. List of glycan probes and their sequences in the screening array

Position[a]	Probe[b]	Sequence
Lactose and N-acetyllactosamine-based		
1	Galactocerebrosides	Galβ-Cer
2	H-Di	Fucα-2Gal-DH
3	A-Tri	GalNAcα-3Gal-DH<br>| Fucα-2
4	B-Tri	Galα-3Gal-DH<br>| Fucα-2
5	B-Tri-AO	Galα-3Gal-AO<br>| Fucα-2
6	GSC-426	(3-deoxy,3-carboxymethyl)Galβ-C30
7	Sulfatide	SU-3Galβ-Cer
8	GSF-1	SU-3Galβ-C30
9	GSC-209	GlcAβ-3Galβ-Cer42
10	GSC-210	SU-3GlcAβ-3Galβ-Cer42
11	GSC-187	NeuAcα-3Galβ-C29
12	GSC-40	NeuAcα-(S)-3Galβ-Cer42
13	GSC-230	NeuAcα-8NeuAcα-3Galβ-Cer36
14	GSC-27	NeuAcα-6Galβ-Cer36
15	GSC-144	KDNα-6Galβ-Cer36
16	GSC-13	NeuAcα-(S)-6Galβ-Cer36
17	GSC-72	NeuAcα-(S)-6Galβ-(S)-Cer36
18	GSC-231	NeuAcα-8NeuAcα-6Galβ-Cer36
19	GSC-439	NeuAcα-8NeuAcα-8NeuAcα-6Galβ-Cer36
20	Glucocerebrosides	Glcβ-Cer
21	GSF-19	SU-6Glcβ-C30
22	GSC-60	NeuAcα-6Glcβ-Cer36
23	GSC-9	NeuAcα-(S)-6Glcβ-Cer36
24	GSC-62	NeuAcα-2Glcβ-Cer36

25	GSC-59	NeuAcα-6GlcNAcβ-Cer36
26	GSC-95	NeuAcα-(S)-6GlcNAcβ-Cer36
27	GSC-232	NeuAcα-8NeuAcα-6Glcβ-Cer36
28	Lactocerebrosides	Galβ-4Glcβ-Cer
29	Lac	Galβ-4Glc-DH
30	Lac-AO	Galβ-4Glc-AO
31	GSC-432	(3-deoxy,3-carboxymethyl)Galβ-4Glcβ-C30
32	GSC-296	GlcAβ-3Galβ-4Glcβ-C30
33	GSC-353	SU-3GlcAβ-3Galβ-4Glcβ-C30
34	GalNAcα-3Galβ-4Glc	GalNAcα-3Galβ-4Glc-DH
35	Globotri-AO	Galα-4Galβ-4Glc-AO
36	Ceramide trihexoside	Galα-4Galβ-4Glcβ-Cer
37	Globoside (P-antigen)	GalNAcβ-3Galα-4Galβ-4Glcβ-Cer
38	Forssmann glycolipid	GalNAcα-3GalNAcβ-3Galα-4Galβ-4Glcβ-Cer
39	Fuc(3)-Lac-AO	Galβ-4Glc-AO<br>| Fucα-3
40	GSC-430	(3-deoxy,3-carboxymethyl)Galβ-3Glcβ-C30<br>| Fucα-4
41	GSC-260	3-deoxy,3-carboxymethyl-Galβ-4Glcβ-C30<br>| Fucα-3
42	GSC-150	SU-3Galβ-4Glcβ-C30<br>| Fucα-3
43	GSC-160	SU-3Galβ-4Glcβ-Cer36<br>| Fucα-3
44	NeuAcα-(3')Lac	NeuAcα-3Galβ-4Glc-DH
45	NeuAcα-(3')Lac-AO	NeuAcα-3Galβ-4Glc-AO
46	Neu4,5Ac-(3')Lac	(4-OAc)NeuAcα-3Galβ-4Glc-DH
47	Neu4,5Ac-(3')Lac-AO	(4-OAc)NeuAcα-3Galβ-4Glc-AO
48	GSC-16	NeuAcα-3Galβ-4Glcβ-Cer32
49	GSC-178	NeuAcα-3Galβ-4Glcβ-Cer34
50	GSC-17	NeuAcα-3Galβ-4Glcβ-Cer36

51	GSC-18	NeuAcα-3Galβ-4Glcβ-Cer42
52	GSC-197	KDNα-3Galβ-4Glcβ-Cer28
53	GSC-199	KDNα-3Galβ-4Glcβ-C30
54	GSC-198	KDNα-3Galβ-4Glcβ-Cer34
55	GSC-75	(4-deoxy)NeuAcα-3Galβ-4Glcβ-Cer36
56	GSC-76	(7-deoxy)NeuAcα-3Galβ-4Glcβ-Cer36
57	GSC-77	(8-deoxy)NeuAcα-3Galβ-4Glcβ-Cer36
58	GSC-153	(4,8-deoxy)NeuAcα-3Galβ-4Glcβ-Cer36
59	GSC-51	(9-deoxy)NeuAcα-3Galβ-4Glcβ-Cer36
60	GSC-78	(4-OMe)NeuAcα-3Galβ-4Glcβ-Cer36
61	GSC-79	(9-OMe)NeuAcα-3Galβ-4Glcβ-Cer36
62	GSC-23	(C7)NeuAcα-3Galβ-4Glcβ-Cer36
63	GSC-24	(C8)NeuAcα-3Galβ-4Glcβ-Cer36
64	GSC-50	(C8 diastereoisomer)NeuAcα-3Galβ-4Glcβ-Cer36
65	GSC-229	NeuAcα-8NeuAcα-3Galβ-4Glcβ-Cer36
66	GSC-96	NeuAcα-9NeuAcα-3Galβ-4Glcβ-Cer36
67	GSC-437	NeuAcα-8NeuAcα-8NeuAcα-3Galβ-4Glcβ-Cer36
68	Neuα-(3')Lac	Neuα-3Galβ-4Glc-DH
69	Neuα-(3')Lac-AO	Neuα-3Galβ-4Glc-AO
70	NeuAcα-(6')Lac	NeuAcα-6Galβ-4Glc-DH
71	NeuAcα-(6')Lac-AO	NeuAcα-6Galβ-4Glc-AO
72	GSC-61	NeuAcα-6Galβ-4Glcβ-Cer36
73	GSC-12	NeuAcα-(S)-6Galβ-4Glcβ-Cer36
74	GSC-234	NeuAcα-(S)-6Galβ-(S)-4Glcβ-Cer36
75	GSC-73	NeuAcα-(S)-6Galβ-4Glcβ-(S)-Cer36
76	Neuα-(6')Lac	Neuα-6Galβ-4Glc-DH
77	Neuα-(6')Lac-AO	Neuα-6Galβ-4Glc-AO
78	NeuAcβ-(3')Lac	NeuAcβ-3Galβ-4Glc-DH
79	NeuAcβ-(3')Lac-AO	NeuAcβ-3Galβ-4Glc-AO
80	NeuAcβ-(6')Lac	NeuAcβ-6Galβ-4Glc-DH

81	NeuAcβ-(6')Lac-AO	NeuAcβ-6Galβ-4Glc-AO
82	GSC-161	NeuAcα-3Galβ-4Glcβ-C30<br>Fucα-3
83	GSC-162	NeuAcα-3Galβ-4Glcβ-Cer36<br>Fucα-3
84	LacNAc(1-3)	Galβ-3GlcNAc-DH
85	LacNAc(1-3)-AO	Galβ-3GlcNAc-AO
86	LacNAc	Galβ-4GlcNAc-DH
87	LacNAc-AO	Galβ-4GlcNAc-AO
88	Galα-4Galβ-4GlcNAc	Galα-4Galβ-4GlcNAc-DH
89	SU(3')-LN	SU-3Galβ-4GlcNAc-DH
90	Lea-Tri	Galβ-3GlcNAc-DH<br>Fucα-4
91	Lea-Tri-AO	Galβ-3GlcNAc-AO<br>Fucα-4
92	Lex-Tri	Galβ-4GlcNAc-DH<br>Fucα-3
93	Lex-Tri-AO	Galβ-4GlcNAc-AO<br>Fucα-3
94	Lex-Tri-(Me)AO	Galβ-4GlcNAc-(Me)AO<br>Fucα-3
95	SU(3')-Lea-Tri	SU-3Galβ-3GlcNAc-DH<br>Fucα-4
96	SU(3')-Lex-Tri	SU-3Galβ-4GlcNAc-DH<br>Fucα-3
97	NeuAcα-(3')LN	NeuAcα-3Galβ-4GlcNAc-DH
98	NeuAcα-(3')LN-AO	NeuAcα-3Galβ-4GlcNAc-AO
99	PI-1	NeuAcα-3(6-NAc)Galβ-4GlcNAc-DH
100	PI-1-AO	NeuAcα-3(6-NAc)Galβ-4GlcNAc-AO
101	PI-2	NeuAcα-3(6-NBz)Galβ-4GlcNAc-DH
102	PI-2-AO	NeuAcα-3(6-NBz)Galβ-4GlcNAc-AO

103	NeuAcα-(6')LN	NeuAcα-6Galβ-4GlcNAc-DH
104	NeuAcα-(6')LN-AO	NeuAcα-6Galβ-4GlcNAc-AO
105	Neu5,9Ac-(6')LN	(9-OAc)NeuAcα-6Galβ-4GlcNAc-DH
106	SA(3')-Lea-Tri	NeuAcα-3Galβ-3GlcNAc-DH<br>| Fucα-4
107	SA(3')-Lea-Tri-AO	NeuAcα-3Galβ-3GlcNAc-AO<br>| Fucα-4
108	SA(3')-Lex-Tri	NeuAcα-3Galβ-4GlcNAc-DH<br>| Fucα-3
109	SA(3')-Lex-Tri-AO	NeuAcα-3Galβ-4GlcNAc-AO<br>| Fucα-3
110	GSC-440	NeuAcα-3Galβ-4GlcNAcβ-C30<br>| Fucα-3
111	GSC-512	(4-OAc)NeuAcα-3Galβ-4GlcNAcβ-C30<br>| Fucα-3
112	GSC-513	(9-OAc)NeuAcα-3Galβ-3GlcNAcβ-C30<br>| Fucα-4
113	GSC-511	(9-OAc)NeuAcα-3Galβ-4GlcNAcβ-C30<br>| Fucα-3
Lacto-N-neotetraose and Lacto-N-tetraose-based		
114	GSC-225	(3-deoxy,3-carboxymethyl)Galβ-4GlcNAcβ-3Galβ-Cer36<br>| Fucα-3
115	GSC-236	SU-3Galβ-4GlcNAcβ-3Galβ-C30<br>| Fucα-3
116	GSC-479	NeuAcα-3Galβ-4GlcNAcβ-3Galβ-C30<br>| Fucα-3
117	GSC-105	NeuAcα-3Galβ-4GlcNAcβ-3Galβ-Cer36<br>| Fucα-3
118	GSC-121	NeuAcα-3Galβ-4GlcNAcβ-3Galβ-Cer36<br>| (3-deoxy)Fucα-3

119	GSC-123	NeuAcα-3Galβ-4GlcNAcβ-3Galβ-Cer36<br>(4-deoxy)Fucα-3
120	GSC-133	NeuAcα-3Galβ-4GlcNAcβ-3Galβ-Cer36<br>(2-OMe)Fucα-3
121	GSC-131	NeuAcα-3Galβ-4GlcNAcβ-3Galβ-Cer36<br>Quvα-3
122	GSC-163	NeuAcα-3Galβ-4GlcNAcβ-3Galβ-Cer36<br>Rhaα-3
123	GSC-127	NeuAcα-3Galβ-4GlcNAcβ-3Galβ-Cer36<br>(6-deoxy)Talα-3
124	GSC-341	KDNα-3Galβ-4GlcNAcβ-3Galβ-C30<br>Fucα-3
125	GSC-177	NeuGcα-3Galβ-4GlcNAcβ-3Galβ-Cer36<br>Fucα-3
126	GSC-175	NeuAcα-3(4-deoxy)Galβ-4GlcNAcβ-3Galβ-Cer36<br>Fucα-3
127	GSC-176	NeuAcα-3(6-deoxy)Galβ-4GlcNAcβ-3Galβ-Cer36<br>Fucα-3
128	GSC-257	NeuAcα-3(4,6-deoxy)Galβ-4GlcNAcβ-3Galβ-Cer36<br>Fucα-3
129	DLNN	GlcNAcβ-3Galβ-4Glc-DH
130	LNT	Galβ-3GlcNAcβ-3Galβ-4Glc-DH
131	Paragloboside	Galβ-4GlcNAcβ-3Galβ-4Glcβ-Cer
132	LNnT	Galβ-4GlcNAcβ-3Galβ-4Glc-DH
133	B-like pentaosylceramide	Galα-3Galβ-4GlcNAcβ-3Galβ-4Glcβ-Cer
134	Klaus glycolipid	Galβ-3Galβ-4GlcNAcβ-3Galβ-4Glcβ-Cer
135	GSC-207	GlcAβ-3Galβ-4GlcNAcβ-3Galβ-4Glcβ-C30
136	GSC-191	GlcAβ-3Galβ-4GlcNAcβ-3Galβ-4Glcβ-Cer36
137	GSC-189	GlcAβ-3Galβ-4GlcNAcβ-3Galβ-4Glcβ-Cer42
138	SU(3')-Tri	SU-3Galβ-4GlcNAcβ-3Gal-DH
139	GSC-208	SU-3GlcAβ-3Galβ-4GlcNAcβ-3Galβ-4Glcβ-C30

140	GSC-192	SU-3GlcAβ-3Galβ-4GlcNAcβ-3Galβ-4Glcβ-Cer36
141	GSC-190	SU-3GlcAβ-3Galβ-4GlcNAcβ-3Galβ-4Glcβ-Cer42
142	Led-II pentaosylceramide	Fucα-2Galβ-3GlcNAcβ-3Galβ-4Glcβ-CerA
143	Led-I pentaosylceramide	Fucα-2Galβ-3GlcNAcβ-3Galβ-4Glcβ-CerB
144	LNFP-I	Fucα-2Galβ-3GlcNAcβ-3Galβ-4Glc-DH
145	B-hexaosylceramide	Galα-3Galβ-4GlcNAcβ-3Galβ-4Glcβ-Cer<br>Fucα-2
146	A-Hexa	GalNAcα-3Galβ-3GlcNAcβ-3Galβ-4Glc-DH<br>Fucα-2
147	A-Hepta	Fucα-4<br>GalNAcα-3Galβ-3GlcNAcβ-3Galβ-4Glc-DH<br>Fucα-2
148	LNFP-II	Galβ-3GlcNAcβ-3Galβ-4Glc-DH<br>Fucα-4
149	LNDFH-II	Galβ-3GlcNAcβ-3Galβ-4Glc-DH<br>Fucα-4 Fucα-3
150	Leb-hexaosylceramide	Fucα-2Galβ-3GlcNAcβ-3Galβ-4Glcβ-Cer<br>Fucα-4
151	LNDFH-I	Fucα-2Galβ-3GlcNAcβ-3Galβ-4Glc-DH<br>Fucα-4
152	LNTFH-I	Fucα-2Galβ-3GlcNAcβ-3Galβ-4Glc-DH<br>Fucα-4 Fucα-2
153	LNFP-III	Galβ-4GlcNAcβ-3Galβ-4Glc-DH<br>Fucα-3
154	LNFP-III-AO	Galβ-4GlcNAcβ-3Galβ-4Glc-AO<br>Fucα-3
155	LNnDFH-I	Fucα-2Galβ-4GlcNAcβ-3Galβ-4Glc-DH<br>Fucα-3
156	LNnDFH-II	Galβ-4GlcNAcβ-3Galβ-4Glc-DH<br>Fucα-3 Fucα-3

157	LNnDFH-V	Galβ-4GlcNAcβ-3Galβ-4Glc-DH<br>Fucα-3 Fucα-2
158	LNnTFH-I	Fucα-2Galβ-4GlcNAcβ-3Galβ-4Glc-DH<br>Fucα-3 Fucα-2
159	SU(3')-LNFP-II	SU-3Galβ-3GlcNAcβ-4Galβ-4Glc-DH<br>Fucα-4
160	SU(6')-LNFP-II	SU-6Galβ-3GlcNAcβ-3Galβ-4Glc-DH<br>Fucα-4
161	SU(3')-LNFP-III	SU-3Galβ-4GlcNAcβ-3Galβ-4Glc-DH<br>Fucα-3
162	SU(6')-LNFP-III	SU-6Galβ-4GlcNAcβ-3Galβ-4Glc-DH<br>Fucα-3
163	SU(3',6)-LNFP-III	SU-6<br>SU-3Galβ-4GlcNAcβ-3Galβ-4Glc-DH<br>Fucα-3
164	LSTa	NeuAcα-3Galβ-3GlcNAcβ-3Galβ-4Glc-DH
165	GSC-272	NeuAcα-3Galβ-3GlcNAcβ-3Galβ-4Glcβ-C30
166	GSC-147	KDNα-3Galβ-3GlcNAcβ-3Galβ-4Glcβ-Cer36
167	GSC-396	NeuGcα-3Galβ-3GlcNAcβ-3Galβ-4Glcβ-C30
168	LSTb	Galβ-3GlcNAcβ-3Galβ-4Glc-DH<br>NeuAcα-6
169	GSC-397	NeuGcα-6Galβ-3GlcNAcβ-3Galβ-4Glcβ-C30
170	DSLNT	NeuAcα-3Galβ-3GlcNAcβ-3Galβ-4Glc-DH<br>NeuAcα-6
171	Sialylparagloboside	NeuAcα-3Galβ-4GlcNAcβ-3Galβ-4Glcβ-Cer
172	GSC-273	NeuAcα-3Galβ-4GlcNAcβ-3Galβ-4Glcβ-C30
173	GSC-31	NeuAcα-3Galβ-4GlcNAcβ-3Galβ-4Glcβ-Cer36
174	LSTc	NeuAcα-6Galβ-4GlcNAcβ-3Galβ-4Glc-DH
175	GSC-516B	Neuα-3Galβ-4GlcNAcβ-3Galβ-4Glcβ-Cer36<br>SU-6

176	SA(3/6)LNFP-I	NeuAcα-3/6Galβ-3GlcNAcβ-3Galβ-4Glc-DH<br>| Fucα-2
177	SA(3')-LNFP-II	NeuAcα-3Galβ-3GlcNAcβ-3Galβ-4Glc-DH<br>| Fucα-4
178	SA(6')-LNFP-VI	NeuAcα-6Galβ-4GlcNAcβ-3Galβ-4Glc-DH<br>| Fucα-3
179	GSC-533	NeuAcα-3Galβ-4GlcNβ-3Galβ-4Glcβ-Cer36<br>| Fucα-3
180	GSC-64	NeuAcα-3Galβ-4GlcNAcβ-3Galβ-4Glcβ-Cer36<br>| Fucα-3
181	SA(3')-LNFP-III	NeuAcα-3Galβ-4GlcNAcβ-3Galβ-4Glc-DH<br>| Fucα-3
182	GSC-472	Neuα-3Galβ-4GlcNAcβ-3Galβ-4Glcβ-Cer36<br>| Fucα-3
183	GSC-97	NeuAcα-6Galβ-4GlcNAcβ-3Galβ-4Glcβ-Cer36<br>| Fucα-3
184	GSC-314	KDNα-3Galβ-4GlcNAcβ-3Galβ-4Glcβ-C30<br>| Fucα-3
185	GSC-149	KDNα-3Galβ-4GlcNAcβ-3Galβ-4Glcβ-Cer36<br>| Fucα-3
186	GSC-311	KDNα-3Galβ-4GlcNAcβ-3Galβ-4Glcβ-C30<br>| Rhaα-3
187	GSC-268	SU-6<br>| NeuAcα-3Galβ-4GlcNAcβ-3Galβ-4Glcβ-Cer36<br>| Fucα-3
188	GSC-268 deNAc	SU-6<br>| Neuα-3Galβ-4GlcNβ-3Galβ-4Glcβ-Cer36<br>| Fucα-3
189	GSC-269	SU-6<br>| NeuAcα-3Galβ-4GlcNAcβ-3Galβ-4Glcβ-Cer36<br>| Fucα-3

190	GSC-406	SU-6<br>| Neuα-3Galβ-4GlcNAcβ-3Galβ-4Glcβ-Cer36<br>| Fucα-3
191	GSC-270	SU-6 SU-6<br>| | NeuAcα-3Galβ-4GlcNAcβ-3Galβ-4Glcβ-Cer36<br>| Fucα-3
Polylactosamine Ii-active		
192	pLNnH	Galβ-4GlcNAcβ-3Galβ-4GlcNAcβ-3Galβ-4Glc-DH
193	GSC-216	GlcAβ-3Galβ-4GlcNAcβ-3Galβ-4GlcNAcβ-3Galβ-4Glcβ-Cer42
194	GSC-217	SU-3GlcAβ-3Galβ-4GlcNAcβ-3Galβ-4GlcNAcβ-3Galβ-4Glcβ-Cer42
195	GSC-218	GlcAβ-3Galβ-4GlcNAcβ-3Galβ-4GlcNAcβ-3Galβ-4Glcβ-Cer36
196	GSC-219	SU-3GlcAβ-3Galβ-4GlcNAcβ-3Galβ-4GlcNAcβ-3Galβ-4Glcβ-Cer36
197	LNH	Galβ-4GlcNAcβ-6<br>| Galβ-4Glc-DH<br>| Galβ-3GlcNAcβ-3
198	iLNO	Galβ-3GlcNAcβ-3Galβ-4GlcNAcβ-6<br>| Galβ-4Glc-DH<br>| Galβ-3GlcNAcβ-3
199	LND	Galβ-4GlcNAcβ-6<br>| Galβ-4GlcNAcβ-6<br>| | Galβ-3GlcNAcβ-3 Galβ-4Glc-DH<br>| Galβ-3GlcNAcβ-3
200	LNnH	Galβ-4GlcNAcβ-6<br>| Galβ-4Glc-DH<br>| Galβ-4GlcNAcβ-3

201	Nonaosylceramide	GlcNAcβ-6<br>GlcNAcβ-6 Galβ-4GlcNAcβ-3Galβ-4Glcβ-Cer<br>Galβ-4GlcNAcβ-3<br>GlcNAcβ-3
202	I-octaosylceramide	Galβ-4GlcNAcβ-6<br>Galβ-4GlcNAcβ-3Galβ-4Glcβ-Cer<br>Galβ-4GlcNAcβ-3
203	I-dodecaosylceramide	Galβ-4GlcNAcβ-6<br>Galβ-4GlcNAcβ-6 Galβ-4GlcNAcβ-3Galβ-4Glcβ-Cer<br>Galβ-4GlcNAcβ-3<br>Galβ-4GlcNAcβ-3
204	I-hexadecaosylceramide	Galβ-4GlcNAcβ-6<br>Galβ-4GlcNAcβ-6 Galβ-4GlcNAcβ-3Galβ-4Glcβ-Cer<br>Galβ-4GlcNAcβ-6 Galβ-4GlcNAcβ-3<br>Galβ-4GlcNAcβ-3<br>Galβ-4GlcNAcβ-3
205	I-eicosaosylceramide	Galβ-4GlcNAcβ-6<br>Galβ-4GlcNAcβ-6 Galβ-4GlcNAcβ-3Galβ-4Glcβ-Cer<br>Galβ-4GlcNAcβ-6 Galβ-4GlcNAcβ-3<br>Galβ-4GlcNAcβ-6 Galβ-4GlcNAcβ-3<br>Galβ-4GlcNAcβ-3<br>Galβ-4GlcNAcβ-3

206	B-like decaosylceramide	Galα-3Galβ-4GlcNAcβ-6<br>Galβ-4GlcNAcβ-3Galβ-4Glcβ-Cer<br>Galα-3Galβ-4GlcNAcβ-3
207	B-like pentadecaosylceramide	Galα-3Galβ-4GlcNAcβ-6<br>Galα-3Galβ-4GlcNAcβ-6 Galβ-4GlcNAcβ-3Galβ-4Glcβ-Cer<br>Galβ-4GlcNAcβ-3<br>Galα-3Galβ-4GlcNAcβ-3
208	B-like eicosaosylceramide	Galα-3Galβ-4GlcNAcβ-6<br>Galα-3Galβ-4GlcNAcβ-6 Galβ-4GlcNAcβ-3Galβ-4Glcβ-Cer<br>Galα-3Galβ-4GlcNAcβ-6 Galβ-4GlcNAcβ-3<br>Galβ-4GlcNAcβ-3<br>Galα-3Galβ-4GlcNAcβ-3
209	B-like pentaeicosaosylceramide	Galα-3Galβ-4GlcNAcβ-6<br>Galα-3Galβ-4GlcNAcβ-6 Galβ-4GlcNAcβ-3Galβ-4Glcβ-Cer<br>Galα-3Galβ-4GlcNAcβ-6 Galβ-4GlcNAcβ-3<br>Galα-3Galβ-4GlcNAcβ-6 Galβ-4GlcNAcβ-3<br>Galβ-4GlcNAcβ-3<br>Galα-3Galβ-4GlcNAcβ-3
210	pLNFH-IV	Galβ-3GlcNAcβ-3Galβ-4GlcNAcβ-3Galβ-4Glc-DH<br>Fucα-3
211	DFpLNH-II	Galβ-3GlcNAcβ-3Galβ-4GlcNAcβ-3Galβ-4Glc-DH<br>Fucα-4 Fucα-3

212	TFpLNH-I	Fucα-2Galβ-3GlcNAcβ-3Galβ-4GlcNAcβ-3Galβ-4Glc-DH<br>Fucα-4 Fucα-3
213	MFLNH-III	Galβ-4GlcNAcβ-6<br>Fucα-3 Galβ-4Glc-DH<br>Galβ-3GlcNAcβ-3
214	DFLNH(b)	Galβ-4GlcNAcβ-6<br>Fucα-3 Galβ-4Glc-DH<br>Galβ-3GlcNAcβ-3<br>Fucα-4
215	DFLNH(c)	Galβ-4GlcNAcβ-6<br>Galβ-4Glc-DH<br>Fucα-2Galβ-3GlcNAcβ-3<br>Fucα-4
216	DFLNH(a)	Galβ-4GlcNAcβ-6<br>Fucα-3 Galβ-4Glc-DH<br>Fucα-2Galβ-3GlcNAcβ-3
217	TFLNH	Galβ-4GlcNAcβ-6<br>Fucα-3 Galβ-4Glc-DH<br>Fucα-2Galβ-3GlcNAcβ-3<br>Fucα-4
218	MFiLNO-IV	Galβ-3GlcNAcβ-3Galβ-4GlcNAcβ-6<br>Fucα-3 Galβ-4Glc-DH<br>Galβ-3GlcNAcβ-3

219	TFiLNO	Galβ-3GlcNAcβ-3Galβ-4GlcNAcβ-6<br>Fucα-4 Fucα-3 Galβ-4Glc-DH<br>Galβ-3GlcNAcβ-3<br>Fucα-4
220	MFLND	Galβ-4GlcNAcβ-6<br>Fucα-3 Galβ-4GlcNAcβ-6<br>Galβ-3GlcNAcβ-3 Galβ-4Glc-DH<br>Galβ-3GlcNAcβ-3
221	MFLNnH(a)	Galβ-4GlcNAcβ-6<br>Fucα-3 Galβ-4Glc-DH<br>Galβ-4GlcNAcβ-3
222	DFLNnH	Galβ-4GlcNAcβ-6<br>Fucα-3 Galβ-4Glc-DH<br>Galβ-4GlcNAcβ-3<br>Fucα-3
223	B-III dodecaosylceramide	Galα-3Galβ-4GlcNAcβ-6<br>Fucα-2 Galβ-4GlcNAcβ-3Galβ-4Glcβ-Cer<br>Galα-3Galβ-4GlcNAcβ-3<br>Fucα-2
224	B-IV tetradecaosylceramide	Galα-3Galβ-4GlcNAcβ-6<br>Fucα-2 Galβ-4GlcNAcβ-3Galβ-4Glcβ-Cer<br>Galα-3Galβ-4GlcNAcβ-3Galβ-4GlcNAcβ-3<br>Fucα-2
225	MSLNH	NeuAcα-6Galβ-4GlcNAcβ-6<br>Galβ-4Glc-DH<br>Galβ-3GlcNAcβ-3

226	MSLNnH-I	Galβ-4GlcNAcβ-6<br>| Galβ-4Glc-DH<br>| NeuAcα-6Galβ-3GlcNAcβ-3
227	DSLNnH	NeuAcα-6Galβ-4GlcNAcβ-6<br>| Galβ-4Glc-DH<br>| NeuAcα-6Galβ-4GlcNAcβ-3
228	MSMFLNH	Galβ-4GlcNAcβ-6<br>| | Fucα-3 Galβ-4Glc-DH<br>| NeuAcα-3Galβ-3GlcNAcβ-3
229	MFMSLNnH	Galβ-4GlcNAcβ-6<br>| | Fucα-3 Galβ-4Glc-DH<br>| NeuAcα-6Galβ-3GlcNAcβ-3
230	GSC-221	NeuAcα-3Galβ-4GlcNAcβ-3Galβ-4GlcNAcβ-3Galβ-4Glcβ-Cer36<br>| Fucα-3
231	GSC-220	NeuAcα-3Galβ-4GlcNAcβ-3Galβ-4GlcNAcβ-3Galβ-4Glcβ-Cer36<br>| | Fucα-3 Fucα-3
232	C4U	NeuAcα-3Galβ-4GlcNAcβ-3Galβ-3GlcNAc-DH<br>| | | SU-6 SU-6 SU-6
N-glycans		
233	Man2(α2)	Manα-2Man-DH
234	Man2(α3)	Manα-3Man-DH
235	Man2(α6)	Manα-6Man-DH
236	Man3(α3,α6)	Manα-6Man-DH<br>| Manα-3
237	Man5(α3,α6)	Manα-3<br>| Manα-6Manα-6Man-DH<br>| Manα-3
238	Man1GN1	Manβ-4GlcNAc-DH
239	Man2GN1	Manα-3Manβ-4GlcNAc-DH

<table>
<tr><td>240</td><td>Man2aGN2</td><td>Manα-6Manβ-4GlcNAcβ-4GlcNAc-DH</td></tr>
<tr><td>241</td><td>Man3GN2</td><td>Manα-6
|
Manβ-4GlcNAcβ-4GlcNAc-DH
|
Manα-3</td></tr>
<tr><td>242</td><td>Man4aGN2</td><td>Manα-3Manα-6
|
Manβ-4GlcNAcβ-4GlcNAc-DH
|
Manα-3</td></tr>
<tr><td>243</td><td>Man4bGN2</td><td>Manα-6
|
Manα-3Manα-6
|
Manβ-4GlcNAcβ-4GlcNAc-DH</td></tr>
<tr><td>244</td><td>Man5GN2</td><td>Manα-6
|
Manα-3Manα-6
|
Manβ-4GlcNAcβ-4GlcNAc-DH
|
Manα-3</td></tr>
<tr><td>245</td><td>Man6GN2</td><td>Manα-6
|
Manα-3Manα-6
|
Manβ-4GlcNAcβ-4GlcNAc-DH
|
Manα-2Manα-3</td></tr>
<tr><td>246</td><td>Man7(D1)GN2</td><td>Manα-6
|
Manα-3Manα-6
|
Manβ-4GlcNAcβ-4GlcNAc-DH
|
Manα-2Manα-2Manα-3</td></tr>
<tr><td>247</td><td>Man7(D1)GN2-AO</td><td>Manα-6
|
Manα-3Manα-6
|
Manβ-4GlcNAcβ-4GlcNAc-AO
|
Manα-2Manα-2Manα-3</td></tr>
</table>

248	Man7(D3)GN2	Manα-2Manα-6<br>Manα-3Manα-6<br>Manβ-4GlcNAcβ-4GlcNAc-DH<br>Manα-2Manα-3
249	Man8(D1D3)GN2	Manα-2Manα-6<br>Manα-3Manα-6<br>Manβ-4GlcNAcβ-4GlcNAc-DH<br>Manα-2Manα-2Manα-3
250	Man9GN2	Manα-2Manα-6<br>Manα-2Manα-3Manα-6<br>Manβ-4GlcNAcβ-4GlcNAc-DH<br>Manα-2Manα-2Manα-3
251	Man9GN2-AO	Manα-2Manα-6<br>Manα-2Manα-3Manα-6<br>Manβ-4GlcNAcβ-4GlcNAc-AO<br>Manα-2Manα-2Manα-3
252	Glc1Man9GN2	Manα-2Manα-6<br>Manα-6<br>Manα-2Manα-3 Manβ-4GlcNAcβ-4GlcNAc-DH<br>Glcα-3Manα-2Manα-2Manα-3
253	Glc1Man9GN2-AO	Manα-2Manα-6<br>Manα-6<br>Manα-2Manα-3 Manβ-4GlcNAcβ-4GlcNAc-AO<br>Glcα-3Manα-2Manα-2Manα-3

254	Glc2Man7(D1)GN1-AO	Manα-6<br>Manα-3Manα-6<br>Manβ-4GlcNAc-AO<br>Glcα-3Glcα-3Manα-2Manα-2Manα-3
255	Glc3Man7(D1)GN1-AO	Manα-6<br>Manα-3Manα-6<br>Manβ-4GlcNAc-AO<br>Glcα-2Glcα-3Glcα-3Manα-2Manα-2Manα-3
256	Man3XylGN2	Manα-6<br>Xylβ-2Manβ-4GlcNAcβ-4GlcNAc-DH<br>Manα-3
257	N1	Galβ-4GlcNAcβ-2Manα-6 Fucα-6<br>Manβ-4GlcNAcβ-4GlcNAc-DH<br>Manα-3
258	N2	Manα-6<br>Manβ-4GlcNAcβ-4GlcNAc-DH<br>Galβ-4GlcNAcβ-2Manα-3
259	N4	Galβ-4GlcNAcβ-2Manα-6<br>Manβ-4GlcNAcβ-4GlcNAc-DH<br>Manα-3
260	GlcNac2Man3-AO	GlcNAcβ-2Manα-6<br>Man-AO<br>GlcNAcβ-2Manα-3
261	N3	GlcNAcβ-2Manα-6 Fucα-6<br>Galβ-4 Manβ-4GlcNAcβ-4GlcNAc-DH<br>GlcNAcβ-2Manα-3

262	NGA2	GlcNAcβ-2Manα-6<br>Manβ-4GlcNAcβ-4GlcNAc-DH<br>GlcNAcβ-2Manα-3
263	NGA2B	GlcNAcβ-2Manα-6<br>GlcNAcβ-4Manβ-4GlcNAcβ-4GlcNAc-DH<br>GlcNAcβ-2Manα-3
264	NGA3B	GlcNAcβ-2Manα-6<br>GlcNAcβ-4Manβ-4GlcNAcβ-4GlcNAc-DH<br>GlcNAcβ-4Manα-3<br>GlcNAcβ-2
265	NGA4	GlcNAcβ-6<br>GlcNAcβ-2Manα-6<br>Manβ-4GlcNAcβ-4GlcNAc-DH<br>GlcNAcβ-2Manα-3<br>GlcNAcβ-4
266	NGA5B	GlcNAcβ-2<br>GlcNAcβ-4Manα-6<br>GlcNAcβ-6<br>GlcNAcβ-4Manβ-4GlcNAcβ-4GlcNAc-DH<br>GlcNAcβ-4Manα-3<br>GlcNAcβ-2
267	GNMan5BGN2	Manα-6<br>Manα-3Manα-6<br>GlcNAcβ-4Manβ-4GlcNAcβ-4GlcNAc-DH<br>GlcNAcβ-2Manα-3

268	NA2	Galβ-4GlcNAcβ-2Manα-6<br>Manβ-4GlcNAcβ-4GlcNAc-DH<br>Galβ-4GlcNAcβ-2Manα-3
269	NA3	Galβ-4GlcNAcβ-2Manα-6<br>Manβ-4GlcNAcβ-4GlcNAc-DH<br>Galβ-4GlcNAcβ-4Manα-3<br>Galβ-4GlcNAcβ-2
270	NA4	Galβ-4GlcNAcβ-6<br>Galβ-4GlcNAcβ-2Manα-6<br>Manβ-4GlcNAcβ-4GlcNAc-DH<br>Galβ-4GlcNAcβ-4Manα-3<br>Galβ-4GlcNAcβ-2
271	Fuc-GlcNAc	Fucα-6GlcNAc-DH
272	Man3FGN2	Manα-6 Fucα-6<br>Manβ-4GlcNAcβ-4GlcNAc-DH<br>Manα-3
273	Man3FXylGN2	Manα-6<br>Xylβ-2Manα-4GlcNAcβ-4GlcNAc-DH<br>Manα-3 Fucα-3
274	NGA2F	GlcNAcβ-2Manα-6 Fucα-6<br>Manβ-4GlcNAcβ-4GlcNAc-DH<br>GlcNAcβ-2Manα-3
275	NA2F	Galβ-4GlcNAcβ-2Manα-6 Fucα-6<br>Manβ-4GlcNAcβ-4GlcNAc-DH<br>Galβ-4GlcNAcβ-2Manα-3

276	NA2F-AO	Galβ-4GlcNAcβ-2Manα-6 Fucα-6<br>Manβ-4GlcNAcβ-4GlcNAc-AO<br>Galβ-4GlcNAcβ-2Manα-3
277	NA2FB	Galβ-4GlcNAcβ-2Manα-6 Fucα-6<br>GlcNAcβ-4Manβ-4GlcNAcβ-4GlcNAc-DH<br>Galβ-4GlcNAcβ-2Manα-3
278	NA3-Lex	Galβ-4GlcNAcβ-2Manα-6<br>Fucα-3 Manβ-4GlcNAcβ-4GlcNAc-DH<br>Galβ-4GlcNAcβ-4Manα-3<br>Galβ-4GlcNAcβ-2
279	A2(2-6)	NeuAcα-6Galβ-4GlcNAcβ-2Manα-6<br>Manβ-4GlcNAcβ-4GlcNAc-DH<br>NeuAcα-6Galβ-4GlcNAcβ-2Manα-3
280	AGP-Bi-Ac2	NeuAcα-6Galβ-4GlcNAcβ-2Manα-6<br>Manβ-4GlcNAcβ-4GlcNAc-DH<br>NeuAcα-6Galβ-4GlcNAcβ-2Manα-3
281	AGP-Bi-Gc2	NeuGcα-6Galβ-4GlcNAcβ-2Manα-6<br>Manβ-4GlcNAcβ-4GlcNAc-DH<br>NeuGcα-6Galβ-4GlcNAcβ-2Manα-3
282	AGP-Bi-AcGc	NeuGc(Ac)α-6Galβ-4GlcNAcβ-2Manα-6<br>Manβ-4GlcNAcβ-4GlcNAc-DH<br>NeuAc(Gc)α-6Galβ-4GlcNAcβ-2Manα-3
283	A3	NeuAcα-3Galβ-4GlcNAcβ-2Manα-6<br>Manβ-4GlcNAcβ-4GlcNAc-DH<br>NeuAcα-3Galβ-4GlcNAcβ-4Manα-3<br>NeuAcα-6Galβ-4GlcNAcβ-2

284	A2F(2-3)	NeuAcα-3Galβ-4GlcNAcβ-2Manα-6 Fucα-6<br>| | Manβ-4GlcNAcβ-4GlcNAc-DH<br>| NeuAcα-3Galβ-4GlcNAcβ-2Manα-3
Gangliosides		
285	GM4	NeuAcα-3Galβ-Cer
286	SM3	SU-3Galβ-4Glcβ-Cer
287	Haematoside	NeuAcα-3Galβ-4Glcβ-Cer
288	GM3	NeuAcα-3Galβ-4Glcβ-Cer
289	GM3(Gc)	NeuGcα-3Galβ-4Glcβ-Cer
290	Asialo-GM2	GalNAcβ-4Galβ-4Glcβ-Cer
291	SB2	SU-3GalNAcβ-4Galβ-4Glcβ-Cer<br>| SU-3
292	GM2	GalNAcβ-4Galβ-4Glcβ-Cer<br>| NeuAcα-3
293	GSC-576	GalNAcβ-4Galβ-3Glcβ-C30<br>| NeuAcα-3
294	GSC-108	GalNAcβ-4Galβ-4Glcβ-Cer36<br>| NeuAcα-3
295	GSC-193	GalNAcβ-4Galβ-4Glcβ-Cer36<br>| KDNα-3
296	Asialo-GM1	Galβ-3GalNAcβ-4Galβ-4Glcβ-Cer
297	Asialo-GM1-Tetra	Galβ-3GalNAcβ-4Galβ-4Glc-DH
298	SM1a	Galβ-3GalNAcβ-4Galβ-4Glcβ-Cer<br>| SU-3
299	SB1a	SU-3Galβ-3GalNAcβ-4Galβ-4Glcβ-Cer<br>| SU-3
300	GSC-335	SU-6<br>| NeuAcα-3Galβ-3GalNAcβ-4Galβ-4Glcβ-Cer36
301	GM1	Galβ-3GalNAcβ-4Galβ-4Glcβ-Cer<br>| NeuAcα-3

302	GM1-penta	Galβ-3GalNAcβ-4Galβ-4Glc-DH<br>| NeuAcα-3
303	GM1(Gc)	Galβ-3GalNAcβ-4Galβ-4Glcβ-Cer<br>| NeuGcα-3
304	GM1(Gc)-penta	Galβ-3GalNAcβ-4Galβ-4Glc-DH<br>| NeuGcα-3
305	GD1a	NeuAcα-3Galβ-3GalNAcβ-4Galβ-4Glcβ-Cer<br>| NeuAcα-3
306	GD1a-hexa	NeuAcα-3Galβ-3GalNAcβ-4Galβ-4Glc-DH<br>| NeuAcα-3
307	GalNAc-GD1a(Ac,Gc)	GalNAcβ-4Galβ-3GalNAcβ-4Galβ-4Glcβ-Cer<br>| | NeuGcα-3 NeuAcα-3<br><br>GalNAcβ-4Galβ-3GalNAcβ-4Galβ-4Glcβ-Cer<br>| | NeuAcα-3 NeuGcα-3
308	GSC-195	KDNα-3Galβ-3GalNAcβ-4Galβ-4Glcβ-Cer36<br>| KDNα-3
309	GD3	NeuAcα-8NeuAcα-3Galβ-4Glcβ-Cer
310	GD3-tetra	NeuAcα-8NeuAcα-3Galβ-4Glc-DH
311	GD3-tetra-AO	NeuAcα-8NeuAcα-3Galβ-4Glc-AO
312	GD2	GalNAcβ-4Galβ-4Glcβ-Cer<br>| NeuAcα-8NeuAcα-3
313	GD1b	Galβ-3GalNAcβ-4Galβ-4Glcβ-Cer<br>| NeuAcα-8NeuAcα-3
314	GD1b-DH	Galβ-3GalNAcβ-4Galβ-4Glcβ-DH<br>| NeuAcα-8NeuAcα-3
315	GT1a	NeuAcα-8NeuAcα-3Galβ-3GalNAcβ-4Galβ-4Glcβ-Cer<br>| NeuAcα-3
316	GT1b	NeuAcα-3Galβ-3GalNAcβ-4Galβ-4Glcβ-Cer<br>| NeuAcα-8NeuAcα-3
317	GQ1b	NeuAcα-8NeuAcα-3Galβ-3GalNAcβ-4Galβ-4Glcβ-Cer<br>| NeuAcα-8NeuAcα-3

318	GSC-442	GalNAcβ-4Galβ-4Glcβ-Cer36<br>NeuAcα-6
319	GSC-68	NeuAcα-6Galβ-3GalNAcβ-4Galβ-4Glcβ-Cer36
320	GSC-107	NeuAcα-6Galβ-3GalNAcβ-4Galβ-4Glcβ-Cer36<br>NeuAcα-6
321	GSC-118	NeuAcα-3Galβ-3GalNAcβ-4Galβ-4Glcβ-Cer36<br>NeuAcα-6
O-glycans		
322	GalNAc-Ser	GalNAcα-Ser-DH
323	GalNAc-Thr	GalNAcα-Thr-DH
324	BSM-Di-A1-AO	NeuGcα-6GalNAc-AO
325	BSM-Di-A2-AO	NeuAcα-6GalNAc-AO
326	GalNAcα-3GalNAc	GalNAcα-3GalNAc-DH
327	Galβ-3GalNAc	Galβ-3GalNAc-DH
328	Galβ-3GalNAc-AO	Galβ-3GalNAc-AO
329	Galβ-6GalNAc	Galβ-6GalNAc-DH
330	Galβ-6GalNAc-AO	Galβ-6GalNAc-AO
331	Man-Ser	Manα-Ser-DH
332	Man-Ser-Succ	Manα-Ser-Succ-DH
333	Man-Thr	Manα-Thr-DH
334	Man-Thr-Succ	Manα-Thr-Succ-DH
335	A8/1	GlcNAcα-4Galβ-OX
336	A8/2	SU-6<br>Fucα-3GlcNAcβ-OY
337	A15/1	SU-6GlcNAcβ-OY
338	A15/3	GlcNAcα-4Galβ-3Galβ-OX<br>Fucα-2
339	Notch-1	Fucα-Thr-DH
340	Notch-2	GlcNAcβ-3Fucα-Thr
341	Notch-3	Galβ-4GlcNAcβ-3Fucα-Thr-DH
342	GSC-488	NeuAcα-3Galβ-3GalNAcβ-C30

343	GSC-491	NeuAcα-3Galβ-3(6-deoxy-6-carboxymethyl)GalNAcβ-C30
344	GSC-489	SU-6<br>| NeuAcα-3Galβ-3GalNAcβ-C30
345	DST	NeuAcα-3Galβ-3GalNAc-DH<br>| NeuAcα-6
346	DST-AO	NeuAcα-3Galβ-3GalNAc-AO<br>| NeuAcα-6
347	GSC-490	NeuAcα-3Galβ-3GalNAcβ-C30<br>| NeuAcα-6
348	GlcNAcβ-3Fuc-AO	GlcNAcβ-3Fuc-AO
349	GlcNAcβ1-2Fuc-AO	GlcNAcβ-2Fuc-AO
350	GlcNAcβ1-4Fuc-AO	GlcNAcβ-4Fuc-AO
351	GlcNAcβ-2Man-AO	GlcNAcβ-2Man-AO
Polysialyl		
352	SA2(α8)	NeuAcα-8NeuAc-DH
353	SA3(α8)	NeuAcα-8NeuAcα-8NeuAc-DH
354	SA4(α8)	NeuAcα-8NeuAcα-8NeuAcα-8NeuAc-DH
355	SA5(α8)*	NeuAcα-8NeuAcα-8NeuAcα-8NeuAcα-8NeuAc-DH
356	SA6(α8)*	NeuAcα-8NeuAcα-8NeuAcα-8NeuAcα-8NeuAcα-8NeuAc-DH
357	SA7(α8)*	NeuAcα-8NeuAcα-8NeuAcα-8NeuAcα-8NeuAcα-8NeuAcα-8NeuAc-DH
358	SA8(α8)*	NeuAcα-8NeuAcα-8NeuAcα-8NeuAcα-8NeuAcα-8NeuAc-8NeuAcα-8NeuAc-DH
359	SA9(α8)*	NeuAcα-8NeuAcα-8NeuAcα-8NeuAcα-8NeuAcα-8NeuAc-8NeuAcα-8NeuAc-8NeuAcα-DH
360	SA10(α8)*	NeuAcα-8NeuAcα-8NeuAcα-8NeuAcα-8NeuAcα-8NeuAcα-8NeuAcα-8NeuAcα-8NeuAcα-8NeuAc-DH
Glycosaminoglycans		
361	HA-S4*	GlcAβ-3GlcNAcβ-4GlcAβ-3GlcNAc-DH
362	HA-S14*	GlcAβ-3GlcNAcβ-4GlcAβ-3GlcNAcβ-4GlcAβ-3GlcNAcβ-4GlcAβ-3GlcNAcβ-4GlcAβ-3GlcNAcβ-4GlcAβ-3GlcNAcβ-4GlcAβ-3GlcNAc-DH
363	Hep-Di IS	ΔUA-4GlcNS-DH<br>| | SU-2 | SU-6

364	Hep-Di-IS-AO	ΔUA-4GlcNS-AO<br>SU-2<br>SU-6
365	CSA-4*	ΔUA-3GalNAcβ-4GlcAβ-3GalNAc-DH<br>SU-4 SU-4
366	CSA-14*	ΔUA-3GalNAcβ-4GlcAβ-3GalNAcβ-4GlcAβ-3GalNAcβ-4GlcAβ-3GalNAcβ-4GlcAβ-<br>SU-4 SU-4 SU-4 SU-4<br>-3GalNAcβ-4GlcAβ-3GalNAcβ-4GlcAβ-3GalNAc-DH<br>SU-4 SU-4 SU-4
367	CSB-4*	ΔUA-3GalNAcβ-4IdoAα-3GalNAc-DH<br>SU-4 SU-4
368	CSB-14*	ΔUA-3GalNAcβ-4IdoAα-3GalNAcβ-4IdoAα-3GalNAcβ-4IdoAα-3GalNAcβ-4IdoAα-<br>SU-4 SU-4 SU-4 SU-4<br>-3GalNAcβ-4IdoAα-3GalNAcβ-4IdoAα-3GalNAc-DH<br>SU-4 SU-4 SU-4
369	CSC-4*	ΔUA-3GalNAcβ-4GlcAβ-3GalNAc-DH<br>SU-6 SU-6
370	CSC-14*	ΔUA-3GalNAcβ-4GlcAβ-3GalNAcβ-4GlcAβ-3GalNAcβ-4GlcAβ-3GalNAcβ-4GlcAβ-<br>SU-6 SU-6 SU-6 SU-6<br>-3GalNAcβ-4GlcAβ-3GalNAcβ-4GlcAβ-3GalNAc-DH<br>SU-6 SU-6 SU-6
371	Hep-4-AO*	SU-2<br>ΔUA-4GlcNSα-4IdoAα-4GlcNS-AO<br>SU-6 SU-2 SU-6

372	Hep-14-AO*	SU-2<br>| ΔUA-4GlcNSα-4IdoAα-4GlcNSα-4IdoAα-4GlcNSα-4IdoAα-4GlcNSα-4IdoAα<br>| | | | | | | | SU-6 SU-2 SU-6 SU-2 SU-6 SU-2 SU-6 SU-2<br>-4GlcNSα-4IdoAα-4GlcNSα-4IdoAα-4GlcNS-AO<br>| | | | | SU-6 SU-2 SU-6 SU-2 SU-6
373	HS-S4-AO*	GlcAβ-4GlcNAcα-4GlcAβ-4aMan-AO
Homo-oligomers		
374	GN2-AO	GlcNAcβ-4GlcNAc-AO
375	GN3	GlcNAcβ-4GlcNAcβ-4GlcNAc-DH
376	GN3-AO	GlcNAcβ-4GlcNAcβ-4GlcNAc-AO
377	GN4-AO*	GlcNAcβ-4GlcNAcβ-4GlcNAcβ-4GlcNAc-AO
378	GN5-AO*	GlcNAcβ-4GlcNAcβ-4GlcNAcβ-4GlcNAcβ-4GlcNAc-AO
379	GN7-AO*	GlcNAcβ-4GlcNAcβ-4GlcNAcβ-4GlcNAcβ-4GlcNAcβ-4GlcNAcβ-4GlcNAc-AO
380	GN8-AO*	GlcNAcβ-4GlcNAcβ-4GlcNAcβ-4GlcNAcβ-4GlcNAcβ-4GlcNAcβ-4GlcNAcβ-4GlcNAc-AO
381	Man4(β4)	Manβ-4Manβ-4Manβ-4Man-DH
382	Man6(β4)	Manβ-4Manβ-4Manβ-4Manβ-4Manβ-4Man-DH
383	Xyl5(β4)	Xylβ-4Xylβ-4Xylβ-4Xylβ-4Xyl-DH
384	Xyl6(β4)	Xylβ-4Xylβ-4Xylβ-4Xylβ-4Xylβ-4Xyl-DH
385	Ara6(α5)	Araα-5Araα-5Araα-5Araα-5Araα-5Ara-DH
386	Ara7(α5)	Araα-5Araα-5Araα-5Araα-5Araα-5Araα-5Ara-DH
387	Nigerose-AO	Glcα-3Glc-AO
388	Malto-2-AO	Glcα-4Glc-AO
389	Malto-4-AO	Glcα-4Glcα-4Glcα-4Glc-AO
390	Malto-6-AO	Glcα-4Glcα-4Glcα-4Glcα-4Glcα-4Glc-AO
391	Malto-7-AO	Glcα-4Glcα-4Glcα-4Glcα-4Glcα-4Glcα-4Glc-AO
392	Malto-8-AO*	Glcα-4Glcα-4Glcα-4Glcα-4Glcα-4Glcα-4Glcα-4Glc-AO
393	Malto-9-AO*	Glcα-4Glcα-4Glcα-4Glcα-4Glcα-4Glcα-4Glcα-4Glcα-4Glc-AO
394	Malto-10-AO*	Glcα-4Glcα-4Glcα-4Glcα-4Glcα-4Glcα-4Glcα-4Glcα-4Glcα-4Glc-AO
395	Malto-11-AO*	Glcα-4Glcα-4Glcα-4Glcα-4Glcα-4Glcα-4Glcα-4Glcα-4Glcα-4Glcα-4Glc-AO
396	Malto-12-AO*	Glcα-4Glcα-4Glcα-4Glcα-4Glcα-4Glcα-4Glcα-4Glcα-4Glcα-4Glcα-4Glcα-4Glc-AO

397	Malto-13-AO*	Glcα-4Glcα-4Glcα-4Glcα-4Glcα-4Glcα-4Glcα-4Glcα-4Glcα-4Glcα-4Glcα-4Glcα-4Glc-AO
398	Dext-2-AO	Glcα-6Glc-AO
399	Dext-3-AO	Glcα-6Glcα-6Glc-AO
400	Dext-4-AO	Glcα-6Glcα-6Glcα-6Glc-AO
401	Dext-5-AO*	Glcα-6Glcα-6Glcα-6Glcα-6Glc-AO
402	Dext-6-AO*	Glcα-6Glcα-6Glcα-6Glcα-6Glcα-6Glc-AO
403	Dext-8-AO*	Glcα-6Glcα-6Glcα-6Glcα-6Glcα-6Glcα-6Glcα-6Glc-AO
404	Dext-9-AO*	Glcα-6Glcα-6Glcα-6Glcα-6Glcα-6Glcα-6Glcα-6Glcα-6Glc-AO
405	Dext-10-AO*	Glcα-6Glcα-6Glcα-6Glcα-6Glcα-6Glcα-6Glcα-6Glcα-6Glcα-6Glc-AO
406	Dext-11-AO*	Glcα-6Glcα-6Glcα-6Glcα-6Glcα-6Glcα-6Glcα-6Glcα-6Glcα-6Glcα-6Glc-AO
407	Dext-12-AO*	Glcα-6Glcα-6Glcα-6Glcα-6Glcα-6Glcα-6Glcα-6Glcα-6Glcα-6Glcα-6Glcα-6Glc-AO
408	Dext-13-AO*	Glcα-6Glcα-6Glcα-6Glcα-6Glcα-6Glcα-6Glcα-6Glcα-6Glcα-6Glcα-6Glcα-6Glcα-6Glc-AO
409	Lam-2-AO	Glcβ-3Glc-AO
410	Lam-3-AO	Glcβ-3Glcβ-3Glc-AO
411	Lam-4-AO	Glcβ-3Glcβ-3Glcβ-3Glc-AO
412	Lam-5-AO	Glcβ-3Glcβ-3Glcβ-3Glcβ-3Glc-AO
413	Lam-6-AO*	Glcβ-3Glcβ-3Glcβ-3Glcβ-3Glcβ-3Glc-AO
414	Lam-7-AO	Glcβ-3Glcβ-3Glcβ-3Glcβ-3Glcβ-3Glcβ-3Glc-AO
415	Curd-8-AO*	Glcβ-3Glcβ-3Glcβ-3Glcβ-3Glcβ-3Glcβ-3Glcβ-3Glc-AO
416	Curd-9-AO*	Glcβ-3Glcβ-3Glcβ-3Glcβ-3Glcβ-3Glcβ-3Glcβ-3Glcβ-3Glc-AO
417	Curd-10-AO*	Glcβ-3Glcβ-3Glcβ-3Glcβ-3Glcβ-3Glcβ-3Glcβ-3Glcβ-3Glcβ-3Glc-AO
418	Curd-11-AO*	Glcβ-3Glcβ-3Glcβ-3Glcβ-3Glcβ-3Glcβ-3Glcβ-3Glcβ-3Glcβ-3Glcβ-3Glc-AO
419	Curd-12-AO*	Glcβ-3Glcβ-3Glcβ-3Glcβ-3Glcβ-3Glcβ-3Glcβ-3Glcβ-3Glcβ-3Glcβ-3Glcβ-3Glc-AO
420	Curd-13-AO*	Glcβ-3Glcβ-3Glcβ-3Glcβ-3Glcβ-3Glcβ-3Glcβ-3Glcβ-3Glcβ-3Glcβ-3Glcβ-3Glcβ-3Glc-AO
421	Cello-3-AO	Glcβ-4Glcβ-4Glc-AO
422	Cello-4-AO	Glcβ-4Glcβ-4Glcβ-4Glc-AO
423	Cello-5-AO*	Glcβ-4Glcβ-4Glcβ-4Glcβ-4Glc-AO
424	Cello-6-AO*	Glcβ-4Glcβ-4Glcβ-4Glcβ-4Glcβ-4Glc-AO
425	Cello-7-AO*	Glcβ-4Glcβ-4Glcβ-4Glcβ-4Glcβ-4Glcβ-4Glc-AO
426	Cello-8-AO*	Glcβ-4Glcβ-4Glcβ-4Glcβ-4Glcβ-4Glcβ-4Glcβ-4Glc-AO

427	Cello-9-AO*	Glcβ-4Glcβ-4Glcβ-4Glcβ-4Glcβ-4Glcβ-4Glcβ-4Glcβ-4Glc-AO
428	Cello-10-AO*	Glcβ-4Glcβ-4Glcβ-4Glcβ-4Glcβ-4Glcβ-4Glcβ-4Glcβ-4Glcβ-4Glc-AO
429	Cello-11-AO*	Glcβ-4Glcβ-4Glcβ-4Glcβ-4Glcβ-4Glcβ-4Glcβ-4Glcβ-4Glcβ-4Glcβ-4Glc-AO
430	Cello-12-AO*	Glcβ-4Glcβ-4Glcβ-4Glcβ-4Glcβ-4Glcβ-4Glcβ-4Glcβ-4Glcβ-4Glcβ-4Glcβ-4Glc-AO
431	Cello-13-AO*	Glcβ-4Glcβ-4Glcβ-4Glcβ-4Glcβ-4Glcβ-4Glcβ-4Glcβ-4Glcβ-4Glcβ-4Glcβ-4Glcβ-4Glc-AO
432	Pust-3-AO	Glcβ-6Glcβ-6Glc-AO
433	Pust-4-AO	Glcβ-6Glcβ-6Glcβ-6Glc-AO
434	Pust-5-AO	Glcβ-6Glcβ-6Glcβ-6Glcβ-6Glc-AO
435	Pust-6-AO	Glcβ-6Glcβ-6Glcβ-6Glcβ-6Glcβ-6Glc-AO
436	Pust-7-AO*	Glcβ-6Glcβ-6Glcβ-6Glcβ-6Glcβ-6Glcβ-6Glc-AO
437	Pust-8-AO*	Glcβ-6Glcβ-6Glcβ-6Glcβ-6Glcβ-6Glcβ-6Glcβ-6Glc-AO
Miscellaneous		
438	Gal	Gal-DH
439	Gal-AO	Gal-AO
440	GalNAc	GalNAc-DH
441	GalNAc-AO	GalNAc-AO
442	Glc	Glc-DH
443	Glc-AO	Glc-AO
444	GN	GlcNAc-DH
445	GN-AO	GlcNAc-AO
446	Man-AO	Man-AO
447	Fuc	Fuc-DH
448	Fuc-AO	Fuc-AO
449	NeuAc	NeuAc-DH
450	NeuAc-AO	NeuAc-AO
451	NeuGc	NeuGc-DH
452	NeuGc-AO	NeuGc-AO
453	Rha	Rha-DH
454	Rha-AO	Rha-AO

455	Galα-6Glc-AO	Galα-6Glc-AO
456	(6P)-Glc-AO	P-6Glc-AO
457	(6P)-Man	P-6Man-DH
458	(6P)-Man-AO	P-6Man-AO
459	(6P)-Fructose-AO	P-6Fru-AO
460	SU-Tyr	SU-Tyr-DH
461	SU-Cholesterol	SU-Cholesterol
462	GN-Asn	GlcNAcβ-Asn-DH
463	Xyl3Glc4	Xylα-6<br>| Glcβ-4Glcβ-4Glcβ-4Glc-DH<br>| | Xylα-6 Xylα-6
464	GSC-284	GalNAcβ-6Galβ-4Glcβ-Cer36<br>| NeuAcα-3
465	GSC-575	GalNAcβ-4Galβ-3Galβ-C30<br>| NeuAcα-3
466	GSC-70	NeuAcα-6Galβ-6GalNAcβ-4Galβ-4Glcβ-Cer36
467	GSC-154	NeuAcα-3Galβ-4GlcNAcβ-6Galβ-4Glcβ-Cer36<br>| Fucα-3
468	GSC-446	NeuAcα-3Galβ-4GlcNAcβ-6GalNAcα-3Galβ-4Glc-C30
469	GSC-441	NeuAcα-3Galβ-4GlcNAcβ-6GalNAcα-3Galβ-4Glcβ-C30
470	GSC-384	NeuAcα-3Galβ-4GlcNAcβ-4GalNAcβ-3Galβ-4Glcβ-C30<br>| Fucα-3
471	Glc4(α6,α4,α4)	Glcα-6Glcα-4Glcα-4Glc-DH
472	Glc(α6,α4,α4)-AO	Glcα-6Glcα-4Glcα-4Glc-AO

473	O1-AO	GlcNAcβ-6<br>| Gal-AO<br>| GlcNAcβ-3
474	Rutinose-AO	Rhaα-6Glc-AO

Asterisks that follow the names of certain probes indicate that predominant components are shown.

[a]Pos, Probe position in the binding chart.

[b]The glycan probes are all lipid-linked neoglycolipids (NGLs) or glycosylceramides and are from the collection assembled in the course of research in the Glycosciences Laboratory. DH, NGLs prepared from reducing oligosaccharides by reductive amination with the amino lipid, 1,2-dihexadecyl-*sn*-glycero-3-phosphoethanolamine (DHPE), DH-NGLs; AO, NGLs prepared from reducing oligosaccharides by oxime ligation with an aminooxy (AO) functionalized DHPE; Cer, natural glycolipids with various ceramide moieties; Cer36 and Cer42, synthetic glycolipids with ceramide having a total of 32 and 42 carbon atoms, respectively; C30, a synthetic lipid [2-(tetradecyl)hexadecanol] with 30 carbon atoms. This is a sub-set of a larger array that will be described elsewhere.
