# Supplementary material for: Mannan detecting C-type lectin receptor probes recognise immune epitopes with diverse chemical, spatial and phylogenetic heterogeneity in fungal cell walls
Source: PLoS Pathog. 2020 Jan 30;16(1):e1007927. doi: 10.1371/journal.ppat.1007927 (PMC7012452; doi:10.1371/journal.ppat.1007927)
Supplement: S4 Table — (PDF) [file ppat.1007927.s007.pdf]

S4 Table. Supplementary glycan microarray document based on MIRAGE Glycan Microarray Guidelines (doi:10.3762/mirage.3).

Classification	Guidelines
**1. Sample: Glycan Binding Sample**	
Description of Sample	Sample names: Murine CRD4-7-Fc, human DC-SIGN-Fc, murine dectin-2-Fc, murine dectin-1-Fc.<br>Origin: recombinant<br>Method of preparation:<br>Please see the *Materials and Methods* section in the main text for all the proteins.
Sample modifications	Not relevant.
Assay protocol	Please see *Materials and Methods* section in the main text.
**2. Glycan Library**	
Glycan description for defined glycans	Two glycan microarrays were used:<br>1) The 'Fungal and Bacterial Polysaccharide Array' contained 19 saccharides (polysaccharides or glycoproteins) and one lipid-linked neoglycolipid (NGL) probe. The probe names and the predominant oligosaccharide sequences are in **S2 Table**.<br>The glucan polysaccharides were described previously (IDs 1-11) (Palma et al. Mol. Cell Proteomics. 2015). Some of the saccharides used in the 'Fungal and Bacterial Polysaccharide Array' were from commercial sources or were gifts from individuals including: *S. cerevisiae* mannan (Sigma), *C. albicans* mannan (David Williams, East Tennessee State University), *Aspergillus fumigatus* mannoprotein (Christopher Thornton, University of Exeter), antigens from *Mycobacterium smegmatis* and *M. tuberculosis* (NIH Biodefense and Emerging Infections Research Resources Repository) and glucurono-xylomannan from *Tremella fuciformis* (Elicityl) (Hanashima, S. et al. Chembiochem. 2015).<br>2) A screening microarray of sequence-defined lipid-linked glycan probes. Names and structures are in **S3 Table B**. They consist of 474 probes described earlier (Palma et al., BBRC. 2011). These are a sub-set of a recently generated large screening microarray containing around 900 glycan probes (in-house designation 'Array Sets 42-56', which will be published elsewhere). The NGL probes are from the collection assembled in the course of research in the Glycosciences Laboratory (https://glycosciences.med.ic.ac.uk/glycanLibraryList.html).
Glycan description for	Not relevant.

undefined glycans	
Glycan modifications	Polysaccharides and glycoproteins were not modified.<br>For NGLs, unless otherwise specified these were prepared from reducing oligosaccharides by reductive amination with the amino lipid, 1,2-dihexadecyl-*sn*-glycero-3-phosphoethanolamine [(DHPE) (Chai et al., Methods Enzymol. 2003)]; AO, NGLs prepared from reducing oligosaccharides by oxime ligation with an aminooxy functionalized DHPE [(AOPE) (Liu et al., Chem. Biol. 2007)].<br>For full description on the definition of lipid moieties of the glycan probes please see https://glycosciences.med.ic.ac.uk/docs/lipids.pdf
**3. Printing Surface; e.g., Microarray Slide**	
Description of surface	Nitrocellulose-coated glass microarray slides.
Manufacturer	16-pad UniSart 3D Microarray Slide from Sartorius (Goettingen, Germany)
Custom preparation of surface	Not relevant.
Non-covalent Immobilisation	The saccharides and the lipid-linked probes were immobilized noncovalently onto nitrocellulose-coated glass slides.<br>For construction of the 'Fungal and Bacterial Polysaccharide Array', polysaccharides and glycoproteins were taken up in water, with the exception of curdlan polysaccharide that was solubilised using mild alkaline solution (50mM NaOH) and glucurono-xylomannan solubilised in 150mM NaCl.<br>The lipid-linked oligosaccharide probes were formulated as liposomes by adding carrier lipids, 1,2-dihexanoyl-*sn*-glycero-3-phosphocholine (DHPC) and cholesterol for arraying and non-covalent immobilization on nitrocellulose-coated glass slides (Liu et al., Methods Mol. Biol. 2012).
**4. Arrayer (Printer)**	
Description of Arrayer	Nano-Plotter 2.1 (GeSiM, Radeberg, Germany).
Dispensing mechanism	Non-contact liquid delivery with four dispensing tips.
Glycan deposition	Approximately 0.33 nl was printed per spot.<br>Polysaccharides and glycoproteins were printed at 0.03 and 0.1 ng per spot, and lipid-linked glycan probes at 2 and 5 fmol per spot, all in duplicate.
Printing conditions	The printing solutions were all aqueous-based. Printing was performed at ambient temperature and relative humidity of 58%.

	The printing solutions for the screening arrays of sequence-defined glycans contained 100 pmol/μl of DHPC and cholesterol (both from SIGMA) as lipid carriers in addition to the lipid-linked glycan probes. The concentrations of the glycan probes were 5 and 15 pmol/μl for the 2 and 5 fmol per spot levels, respectively.<br>The printing solutions also contained Cy3 NHS ester (GE Healthcare) at 20 ng/ml (26 fmol/μl) as a marker to monitor the printing process.
**5. Glycan Microarray with "Map"**	
Array layout	Each array slide contained 16-pad subarrays. Each pad was set up for printing 64 probes maximum, each at 2 levels in duplicate (four spots for one probe in a row); up to 256 spots (16x16) in total in each pad.<br>The 'Fungal and Bacterial Polysaccharide Array' contained 19 probes. The remaining space in each pad was treated as 'blank' when using a grid of 16x16 per pad during the quantitation process.<br>The 474 lipid-linked probes in the screening arrays were printed on multiple subarrays for parallel binding analyses.
Glycan identification and quality control	The 'Fungal, and Bacterial Polysaccharide Array' was previously analysed with Fc-tagged Dectin-1-Fc and a number of anti-Candida mAbs (Rudkin et al., Nature Communications. 2018). For quality control purposes in this study the data of dectin-1-Fc is included in Fig. 10. Other data with sequence-specific proteins, available on request include: (1) monoclonal anti-dextran antibodies and carbohydrate-binding modules of bacterial glycoside hydrolases with specificity for α-glucans (TmCBM41) and β-glucans (CmCBM6-2, CtCBM11 and CmCBM32-2); these proteins were used in Palma, et al. Mol. Cell Proteomics. 2015; (2) anti-β1,3-glucan and anti-β1,3/β1,4-glucan antibodies (Biosupplies).<br>The quality control of the screening microarrays of sequence-defined glycan probes was carried out with: biotinylated plant lectins - *Ricinus Communis* Agglutinin I (RCA120), *Aleuria aurantia* lectin (AAL), Concanavalin A (ConA) and WGA (Vector Laboratories); sialic-binding simian virus 40 VP1 (Campanero-Rhodes et al., J Virol. 2007); and short fiber knob protein of human adenovirus 52 (Lenman et al., Proc Natl Acad Sci U S A. 2018).<br>These data will be described elsewhere.
**6. Detector and Data Processing**	
Scanning hardware	GenePix 4300A (Molecular Devices, UK)
Scanner settings	Scanning resolution: 10 μm / pixel (this resolution is adequate for the sizes of sample spots)<br>Laser channel: Red (scan wavelength 635 nm)<br>PMT: 350

	Scan powers: 10%, 15%, 30% or 90% to achieve maximum signal without spot saturation.
Image analysis software	GenePix® Pro 7 (Molecular Devices)
Data processing	The gpr file was entered into an in-house microarray database using software (designed by Mark Stoll, http://www.beilstein-institut.de/en/publications/proceedings/glyco-2009) for data processing. No particular normalization method or statistical analysis was used.
**7. Glycan Microarray Data Presentation**	
Data presentation	The microarray binding results are in **Figures 10** and **11** and in **S2-3 Tables.**
**8. Interpretation and Conclusion from Microarray Data**	
Data interpretation	No software or algorithms were used to interpret processed data.
Conclusions	In the 'Fungal, and Bacterial Polysaccharide Array', CRD4-7-Fc and hDC-SIGN-Fc showed similar binding profiles towards *C. albicans* *N*-mannoprotein and other mannan related saccharides, e.g. those from *S. cerevisiae* and *M. Tuberculosis*; whereas dectin-2-Fc did not show detectable binding under the assay conditions tested.<br><br>The control protein dectin-1 showed as predicted binding exclusively to β-1,3-glucan related saccharides.<br><br>In the screening arrays with sequence-defined glycans, hDC-SIGN-Fc gave the strongest overall binding and toward the broadest range of glycans among the three proteins tested; the ligands included oligo/high-mannose *N*-glycans and other mannose containing probes, as well as fucose- and GlcNAc-terminating and glucan-related sequences.<br><br>CRD4-7-Fc bound strongly to oligo/high-mannose *N*-glyans, and to fucose containing glycans such as Fuc-GlcNAc and Man3FGN2 and to β-1,4-oligomannoses.<br><br>Dectin-2-Fc showed the lowest overall binding mainly to high-mannose Man_9GN_2 derived probes. Binding was also detected to 3'sialyl LNFPIII and a number sulphated glycans as with hDC-SIGN-Fc.
